# Microbiota-mediated nitrogen fixation and microhabitat homeostasis in aerial root-mucilage

**DOI:** 10.1186/s40168-023-01525-x

**Published:** 2023-04-21

**Authors:** Zhiqiang Pang, Xinyu Mao, Shaoqun Zhou, Sheng Yu, Guizhou Liu, Chengkai Lu, Jinpeng Wan, Lingfei Hu, Peng Xu

**Affiliations:** 1 CAS Key Laboratory of Tropical Plant Resources and Sustainable Use, Xishuangbanna Tropical Botanical Garden, Chinese Academy of Sciences, Mengla, China; 2 College of Life Sciences, University of Chinese Academy of Sciences, Beijing, China; 3 Center of Economic Botany, Core Botanical Gardens, Chinese Academy of Sciences, Mengla, China; 4 Shenzhen Branch, Guangdong Laboratory of Lingnan Modern Agriculture, Key Laboratory of Synthetic Biology, Ministry of Agriculture and Rural Affairs, Agricultural Genomics Institute at Shenzhen, Chinese Academy of Agricultural Sciences, Shenzhen, China; 5 Institute of Soil and Water Resources and Environmental Science, College of Environmental and Resource Sciences, Zhejiang Provincial Key Laboratory of Agricultural Resources and Environment, Zhejiang University, Hangzhou, China

**Keywords:** Rhizosphere ecology, Mucilage microhabitat, Diazotroph, Friendly microbe, Microbial homeostasis

## Abstract

**Background:**

Plants sustain intimate relationships with diverse microbes. It is well-recognized that these plant-associated microbiota shape individual performance and fitness of host plants, but much remains to be explored regarding how they exert their function and maintain their homeostasis.

**Results:**

Here, using pink lady (*Heterotis rotundifolia*) as a study plant, we investigated the phenomenon of microbiota-mediated nitrogen fixation and elucidated how this process is steadily maintained in the root mucilage microhabitat. Metabolite and microbiota profiling showed that the aerial root mucilage is enriched in carbohydrates and diazotrophic bacteria. Nitrogen isotope-labeling experiments, ^15^N natural abundance, and gene expression analysis indicated that the aerial root-mucilage microbiota could fix atmospheric nitrogen to support plant growth. While the aerial root mucilage is a hotspot of nutrients, we did not observe high abundance of other environmental and pathogenic microbes inside. We further identified a fungus isolate in mucilage that has shown broad-spectrum antimicrobial activities, but solely allows the growth of diazotrophic bacteria. This “friendly” fungus may be the key driver to maintain nitrogen fixation function in the mucilage microhabitat.

Video Abstract

**Conclusion:**

The discovery of new biological function and mucilage-habitat friendly fungi provides insights into microbial homeostasis maintenance of microenvironmental function and rhizosphere ecology.

**Supplementary Information:**

The online version contains supplementary material available at 10.1186/s40168-023-01525-x.

## Introduction

Plant-associated microbes play an important role in host nutrient utilization, stress tolerance, plant health, and adaptation. Among those microbes, symbiotic and associative diazotrophs that can fix nitrogen and directly enhance the nitrogen supply to host plants have attracted much attention. To date, outside the underground root/rhizosphere diazotrophs, special niches such as rhizome, xylem, and aerial root have attracted most of the attention [1–3]. Recently, a pioneering study showed that aerial roots of a Mexican maize cultivar exude a large amount of carbohydrate-rich mucilage, which contributes 29–82% of the plant nitrogen from atmospheric nitrogen [4]. Analysis of the mucilage microbiota indicated that it was enriched in nitrogen diazotrophic bacterial genera such as *Burkholderia*, *Herbaspirillum*, and *Azospirillumin*, suggesting the essential roles of mucilage-microbiota system in fulfilling the nitrogen demand of plant [4]. While the underlying mechanisms of mucilage secretion and nitrogen fixation remain unclear, the carbohydrate-rich mucilage was recognized as a vital zone in mediating maize-diazotrophic microbiota interactions [3, 5]. Strikingly, this nutrient-rich spot specifically enriches nitrogen-fixing bacteria rather than pathogens and environmental microbes [6, 7], suggesting the micro-homeostasis behind. However, the underpinning mechanisms of this process are still unknown.

It is increasingly recognized the plant specialized metabolites in root exudates play important roles in defending against pathogens and maintaining microbial homeostasis in the rhizosphere [8–11]. For instance, root-secreted coumarin can inhibit pathogens and influence the assembly of rhizosphere microbiota [12]. Besides host plant-derived regulators, the interactions among microbiota contribute to the establishment, stability, and resilience of host-associated microbial communities [13]. For instance, the root bacterial *Variovorax* and *Pseudomonas* species can maintain root fungal homeostasis, thereby promoting the survival of their host plant *Arabidopsis thaliana* [14]. In the second line of defense (within the root), enriched endophytic bacterial microbiota, Chitinophagaceae, and Flavobacteriaceae can still become the protective layer of the host plant, functioning as a second line of defense within the root [15]. These “friendly” microbes play an important role in maintaining microbial homeostasis and defending against pathogens in the microenvironment. In addition, host-microbe and microbe-microbe interactions can also simultaneously produce synergistic effects to maintain microbial homeostasis in plant roots, such as Arabidopsis root bacterial microbial communities and plant metabolite tryptophan jointly control soil fungal pathogens *Plectosphaerella cucumerina* to promote plant growth and health [16]. In summary, current hypotheses about plant-associated microbial homeostasis are the result of host plant-microbiota and microbiota-microbiota interactions. To find out the key mucilage compound and friendly microbe as well as its protective association with diazotrophic microbiota is of great value for understanding the microbial homeostasis of the mucilage-microbiota system.

**Fig. 1:**
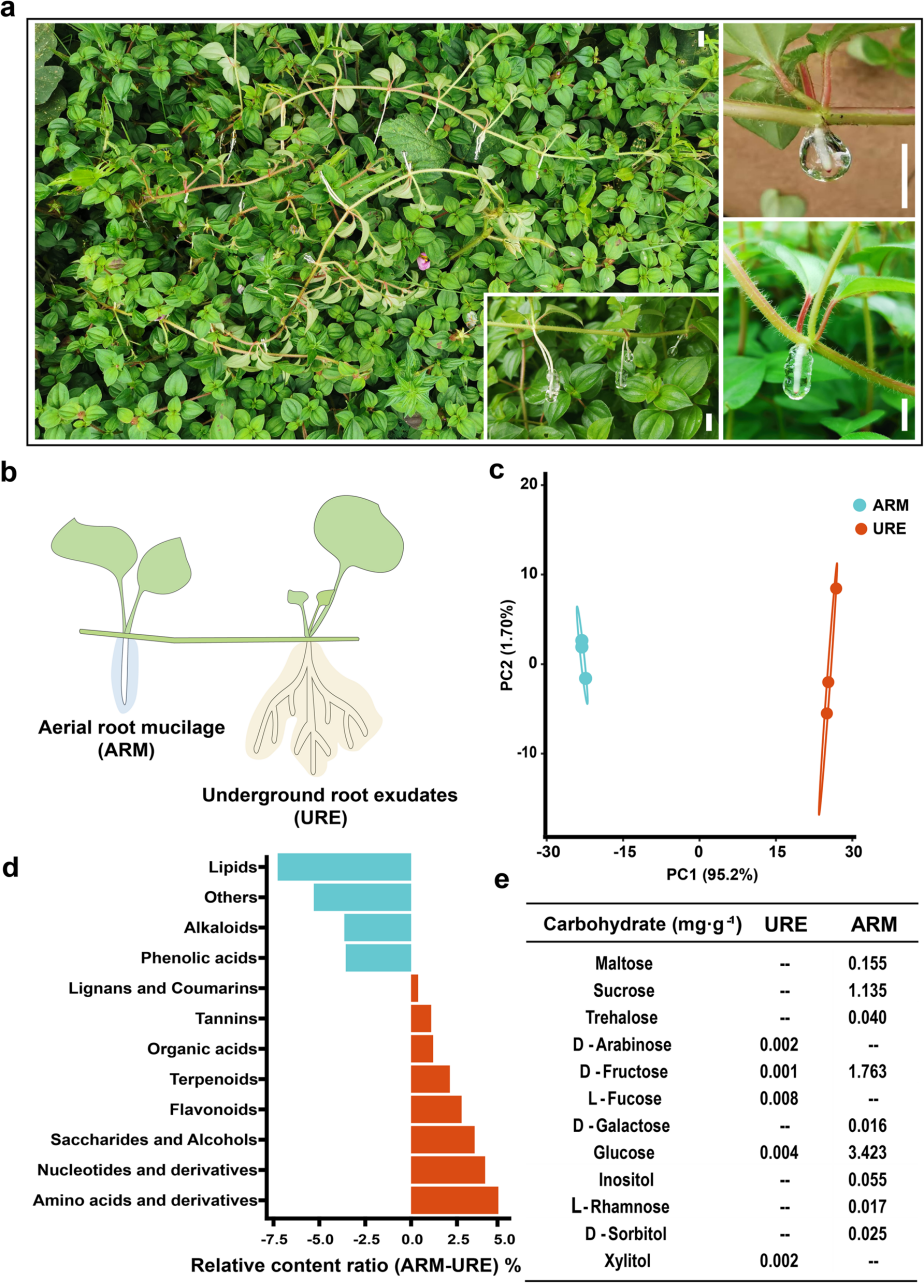
Aerial root mucilage (ARM) and underground root exudate (URE) compound of *H. rotundifolia*. **a** Creeping *H. rotundifolia* with aerial root and mucilage of varying lengths. White scale bars = 1 cm. **b** Sampling diagram of aerial root mucilage and underground root exudates. **c** Principal component analysis result of widely targeted metabolome of ARM and URE. **d** Relative content of different compounds of ARM and URE. Blue indicates that the content of URE is higher than that of ARM, and red indicates that the content of ARM is higher than that of URE. See also Table S1-1. **e** Carbohydrate content of aerial root mucilage and underground root exudate, on average, *n* = 6. See also Table S1-2

Pink lady (*Heterotis rotundifolia*) is a fast-growing, perennial shrub, dicotyledon plant. It is a high-risk invasive plant listed in the Global Compendium of Weeds [17, 18]. Their aerial roots vary from 0.1 to 16 cm and exude a large amount of mucilage before reaching the ground (Fig. 1a). Efficient uptake and utilization of nitrogen by plants seem to contribute to their successful invasion, a process that is accompanied by early reproduction and many offspring and high growth rates [19, 20]. Given the roles of aerial roots in nitrogen uptake stated above, the aerial root-mucilage-microbiota system may contribute to its nitrogen-fixing to achieve their growth and spread. In this study, we use this dicotyledon plant as a model to answer the following two scientific questions: what is the biological function of the aerial root-mucilage system and underlying mechanisms in dicotyledon plants? As a “natural medium,” how does mucilage microhabitat maintain its function and homeostasis in the fluctuant open air?

## Results

### Aerial root mucilage and underground root exudate of *H. rotundifolia* have distinct biochemical composition

The creeping *H. rotundifolia* plants can grow over 2 m long and form the aerial root at each stem node (Fig. 1a). These mucilage-producing aerial roots vary from barely visible to the naked eye to over 16 cm in length. We compared the biochemical composition of the aerial root mucilage (ARM or mucilage) and underground root exudates (URE) by collecting widely targeted metabolomics data of these samples (Fig. 1b) [21]. Principal-component analysis (PCA) results demonstrated a clear differentiation of metabolic profile of ARM and URE samples (Fig. 1c). Indeed, 531 of the 1033 putatively annotated metabolites were significantly different between these two samples (*P* < 0.01, Table S1 and Fig. S1). Whereas URE tended to contain higher levels of lipids and alkaloids, ARM richer in amino acid derivatives, nucleotide derivatives, flavonoids, and carbohydrates (Fig. 1d). Further carbohydrate measurement with a targeted approach confirmed that the ARM was rich in glucose, fructose and sucrose, which were barely detectable in URE (< 0.01 mg·g^−1^; Fig. 1e).

### Aerial root mucilage microbiota of *H. rotundifolia* is enriched in diazotrophic bacteria

**Fig. 2:**
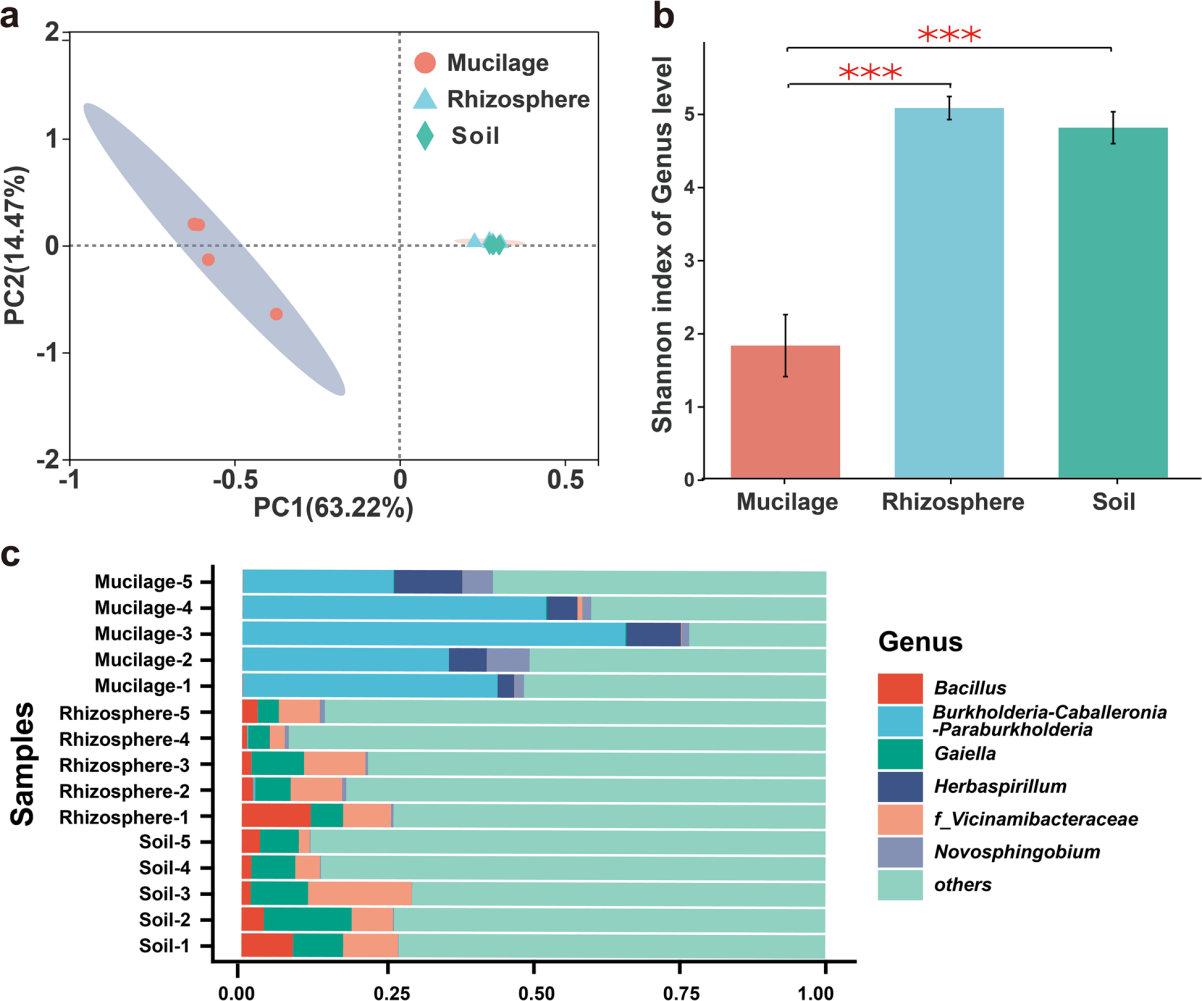
Bacteria diversity and community of aerial root mucilage (mucilage) and underground rhizosphere soil (rhizosphere). **a** Unconstrained PCoA with Bray–Curtis distance showing that the bacteria of mucilage separate from those of rhizosphere and soil in the first axis (*P* < 0.001, permutational multivariate analysis of variance (PERMANOVA) by Adonis). **b** Shannon index of the bacteria of aerial root mucilage, underground rhizosphere soil, and the corresponding bulk soils (*P* < 0.01, two-sided Wilcoxon test). **c** Top three Genus-level distribution of bacterial communities in mucilage and rhizosphere soil. *Burkholderia-Caballeronia-Paraburkholderia*, *Herbaspirillum*, and *Novosphingobium* are higher relative abundance in mucilage (59.09%, 10.43%, and 5.36%, respectively) than in underground rhizosphere soil (0.14%, 0.22%, and 0.64%, respectively). The numbers of replicated samples in this figure are as follows: mucilage (*n* = 5), rhizosphere soil (*n* = 5), and soil (*n* = 5)

As underscored by their biochemical differences, ARM and URE likely represent distinct ecological niches that are suitable to host different microbiota. To test this hypothesis, we collected aerial root mucilage, rhizospheric samples, and environmental soil at five separated sites in Xishuangbanna Tropical Botanical Garden (Xishuangbanna, China) and examined their prokaryotic and eukaryotic microbiota through 16S rRNA and ITS gene sequencing (Table S2 and Fig. S2). In support of our hypothesis, unconstrained principal coordinate analysis (PCoA) based on the Bray–Curtis metric revealed that both the bacterial and fungal composition of mucilage were distinct from those of rhizospheric and bulk soil, which were similar to each other (Fig. 2a bacteria and S2a fungi). Notably, the prokaryotic taxonomic diversity of ARM was lower than rhizospheric and bulk soil, indicative of a specialized bacterial community in ARM (Fig. 2b). For fungal communities, there was significant difference in alpha diversity among the mucilage and bulk soil ecological niches (Fig. S2b, *P* < 0.01). A total of 233 differentially enriched bacterial genera and 65 fungal genera were identified between mucilage and rhizosphere soil (Wilcoxon rank-sum test, *P* < 0.05, Table S3). Specifically, mucilage contained a higher load of *Burkholderia-Caballeronia-Paraburkholderia*, *Herbaspirillum*, and *Novosphingobium*, whereas the relative abundance of *Bacillus*, *Gaiella*, and *Nocardioides* were higher in the underground sample (Fig. 2c and S3a-d).

The top two ARM-enriched bacterial genera, *Burkholderia-Caballeronia-Paraburkholderia* and *Herbaspirillum*, include numerous diazotrophic species that have been widely used as model systems of plant-associated nitrogen-fixing microbes [22, 23]. Some of the single-strain cultures we obtained were identified as *Klebsiella*, *Sphingobacterium*, *Cupriavidus*, *Acinetobacter*, and *Pantoea* species were able to grow on nitrogen-free medium. From these species, we were able to clone different alleles of the key nitrogen fixation gene, *nifH* (Table S4 and Fig. S4a). Most number of these species further demonstrated nitrogen fixation activity in ^15^N_2_-labeled experiments (Fig. S4b). In addition, bacterial function annotation showed that aerial mucilage has a higher nitrogen fixation capacity than rhizosphere soil (Wilcoxon rank-sum test, *P* < 0.05, Fig. S3e, f). These data lead us to hypothesize that nitrogen fixation may be a key function of the bacterial community associated with *H. rotundifolia* aerial root mucilage.

### Aerial root mucilage of *H. rotundifolia* fixes atmospheric nitrogen to support plant growth

**Fig. 3:**
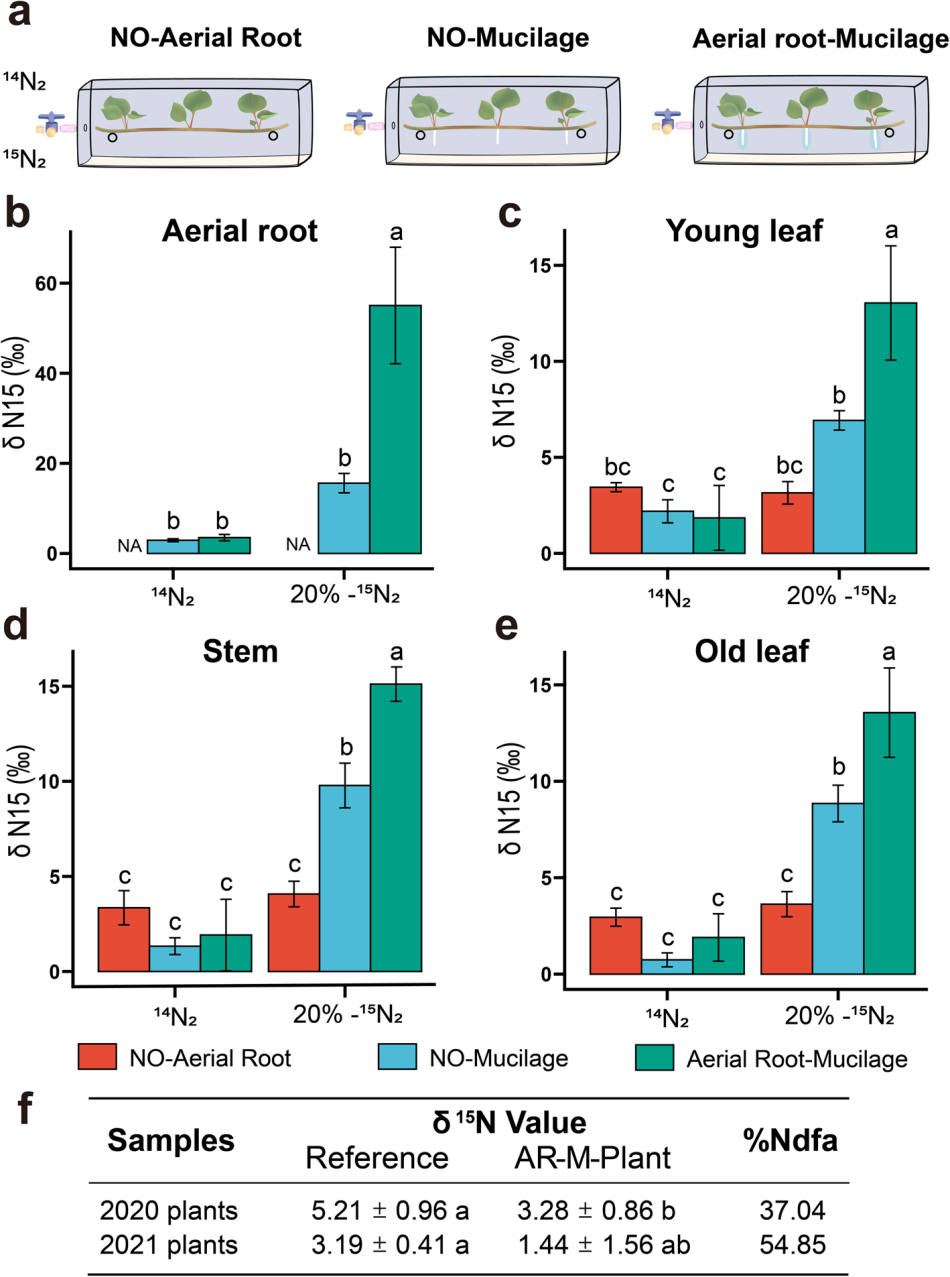
Aerial root-mucilage is the main site for nitrogen fixation in *H. rotundifolia* aerial root. **a** Illustration of the incubation set-up enabling the study of the transfer of aerial mucilage fixed (^15^N-enriched) N_2_ from the air to the plant. The plant types include no aerial root plant (NO-aerial root), aerial root plant without mucilage (NO-mucilage), and aerial root plant with mucilage (aerial root-mucilage). **b**–**e** Analysis of different *H. rotundifolia* samples for ^15^N_2_ enrichment in the aerial root (**e**), young (**d**), old leaf (**f**), and stem (**e**). Box plots show combined data from six independent experiments with natural nitrogen or replacing 20% (V) of nitrogen with ^15^N_2_ for 72 h. ^14^N_2_ and no aerial root plant (NO-aerial root) samples were used as negative control groups. Different letters indicate significantly different groups (*P* < 0.05, two-way ANOVA, *n* = 3). **f** Natural ^15^N abundance values from mucilage-producing plants (AR-M *H. rotundifolia* plant) and reference plants (no aerial root *H. rotundifolia* plant) sampled in XTBG each field. ^15^*N* values are given as mean and standard deviation (*P* < 0.01, *T* test, *n* = 6)

To test if nitrogen fixation could occur in intact ARM microbiota, we performed ^15^N-labeled nitrogen gas (purity specification ≥ 98% and dilution fed to 5%, 10%, and 20% ^15^N_2_ in the bottle, _V_·_V_^−1^) feeding experiments with wild segments of *H. rotundifolia* stems with mucilage-bearing aerial roots, aerial roots with mucilage artificially removed, or aerial roots removed as a whole (Fig. 3a). When fed with 20% ^15^N_2_, plants with mucilage-bearing aerial roots contained significantly higher relative abundance of ^15^N in all tissues measured compared to those with aerial roots only or no aerial roots at all plant tissues (*P* < 0.05, Fig. 3b-e and Table S5). To further verify nitrogen fixation could be utilized by the host plant, we detected the enrichment of ^15^N in plant chlorophyll (converted to pheophytin for analysis) in mucilage-producing plants, but not in the negative controls (Fig. S5). Moreover, the contribution of atmospheric nitrogen fixation to mucilage plants can be quantified by natural ^15^N abundance values of nitrogen-fixing plants and non-fixing (reference) plants. The soil δ^15^N is more abundant than in air; thus, nitrogen-fixing plants will exhibit reduced δ^15^N levels compared to reference non-fixing plants. The samples of mucilage-producing plants and reference plants growing near each other were collected. In 2 years, the natural ^15^N values of leaf and stem for the mucilage-producing samples were lower than the no aerial root plants (Fig. 3f and Table S5), indicating that the aerial root-mucilage system was able to derive a significant part of plant tissue nitrogen from atmospheric nitrogen. The percent of nitrogen derived from the atmosphere (%Ndfa) calculated from the ^15^N natural abundance values in the results ranged from 37.04 to 54.85% (Fig. 3f and Table S5). These results support our hypothesis that intact aerial root-mucilage-microbiota can facilitate incorporation of atmospheric nitrogen for plant. To further test whether the root mucilage system could fulfill *H. rotundifolia* plant nitrogen requirements and growth promotion, ARM-dependent nitrogen fixation is ecologically relevant for the fitness of *H. rotundifolia* in greenhouse and wild, we compared the growth of plants with aerial roots artificially excised versus sympatric intact controls in a field experiment. Four months after root excision (all plant aerial roots not entering soil), plants with aerial roots-mucilage had more than 40% total nitrogen content and 10% greater dry mass than those without, suggesting that the aerial roots, along with the microbiota hosted in their associated mucilage, can play non-trivial roles in fixing nitrogen and supporting the growth of *H. rotundifolia* (Table S5).

### Tissue-specific gene expression in mucilage-secreting aerial roots could facilitate efficient carbon–nitrogen exchange with mucilage microbiota

Nitrogen fixation is a complex symbiotic relationship that requires extensive collaboration between the host plants and the diazotrophs at molecular level. To investigate the molecular interaction between *H. rotundifolia* and its ARM microbiota, we first sequenced the *H. rotundifolia* genome with a combination of 61.86 G PacBio CLR reads and 20.1 G Hi-C reads to obtain a chromosome-scale reference genome assembly, which was subsequently used to map short-read RNAseq data for transcriptome-wide gene expression profiling. The reference assembly we obtained was estimated to be 171.44 Mb, similar to our previous estimation (180 M, Fig. S6a). We annotated 29,574 putative gene models and 2434 noncoding RNAs (Table S6).

**Fig. 4:**
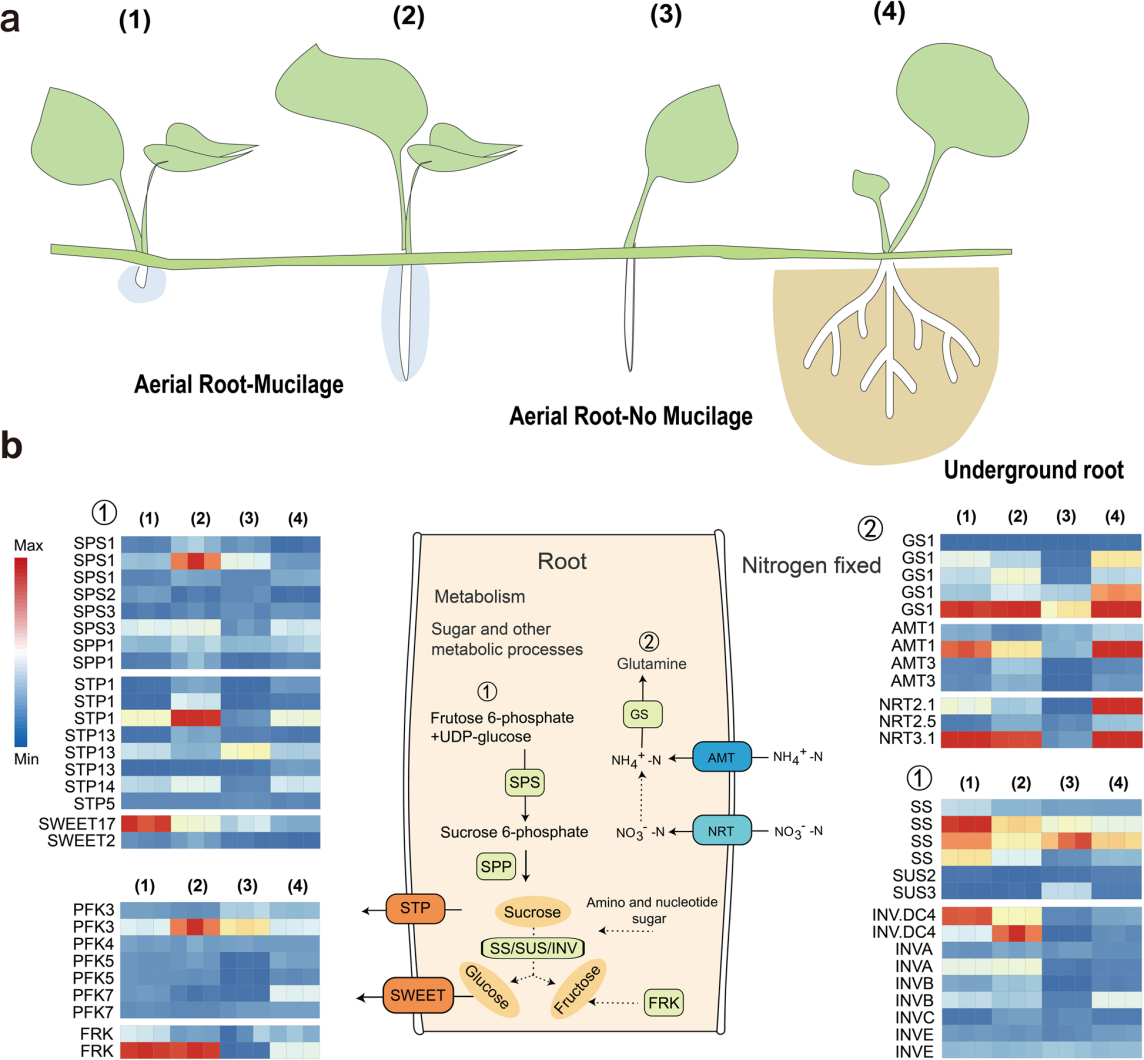
Transcriptome of *H. rotundifolia* tissues reveals the biological function of aerial root-mucilage. **a** Overview of samples aerial root-mucilage and no mucilage and underground root of *H. rotundifolia*. (1–2) Aerial roots with mucilage. (3) Aerial roots without mucilage. (4) Underground root sample without mucilage. **b** Expression patterns of nitrogen uptake and assimilation genes and plant metabolism genes in the different root types. ① and ② are overview of pathways of sugar transport and nitrogen utilization. Genes with expression FPKM < 10 were not visualized in the heatmaps

We then obtained RNA-seq data from aerial roots with or without mucilage and underground roots to identify transcriptome-wide gene expression differences in these tissues (Fig. 4a). Functional enrichment analysis results based on GO and KEGG annotations revealed that genes related to photosynthesis and phenylpropanoid metabolism were preferentially expressed in aerial roots, and these differences in gene expression were more pronounced when comparing mucilage-bearing aerial roots and underground roots (Table S7; Fig. S6d-e). Stable plant-diazotroph symbiosis is built upon extensive carbon flow from the host plant to the microbes, accompanied by nitrogen flow in reverse [3, 24]. Hence, we specifically examined the expression of carbohydrate and accessible nitrogen transporter genes in the four tissues. In result, expression of both putative sugar transporters (e.g.,* STP, SWEET, INV*) and nitrate/nitrite transporters and assimilation genes (e.g.,* AMT*, *NRT,* and *GS*) were significantly higher in mucilage-bearing aerial roots than those without mucilage, indicative of more active carbon–nitrogen flow when the mucilage is present (Fig. 4b). Interestingly, the putative nitrate and nitrite transporters highly expressed in mucilage-bearing aerial roots were also highly expressed in underground roots (with the notable exception of *NRT2.1*), whereas the putative carbohydrate transporters were expressed at higher levels in mucilage-bearing aerial roots than underground roots. *H. rotundifolia* as the first sequenced genome of this genus plant and mucilage-producing dicotyledon, provides the molecular basis for further studies on mucilage exudation and nitrogen fixation mechanism.

### Mucilage microhabitat dwelling *Chaetomella raphigera* selectively inhibit environmental but not sympatric diazotrophic microbes

Though the carbohydrate-rich mucilage of *H. rotundifolia* creates an ideal niche and “natural medium” for diazotrophic bacteria, it is also potentially prone to disturbance by environmental microbes that are commensal or even pathogenic to host plants. Though there is a rich literature on the microbiota structuring function of exuded plant specialized metabolites [13, 16, 25, 26], we were not able to identify any specific compound that displayed obvious antibiotic function when screening the twenty most abundant metabolites in the mucilage at physiologically relevant concentration (Fig. S7 and Table S8-9).

**Fig. 5:**
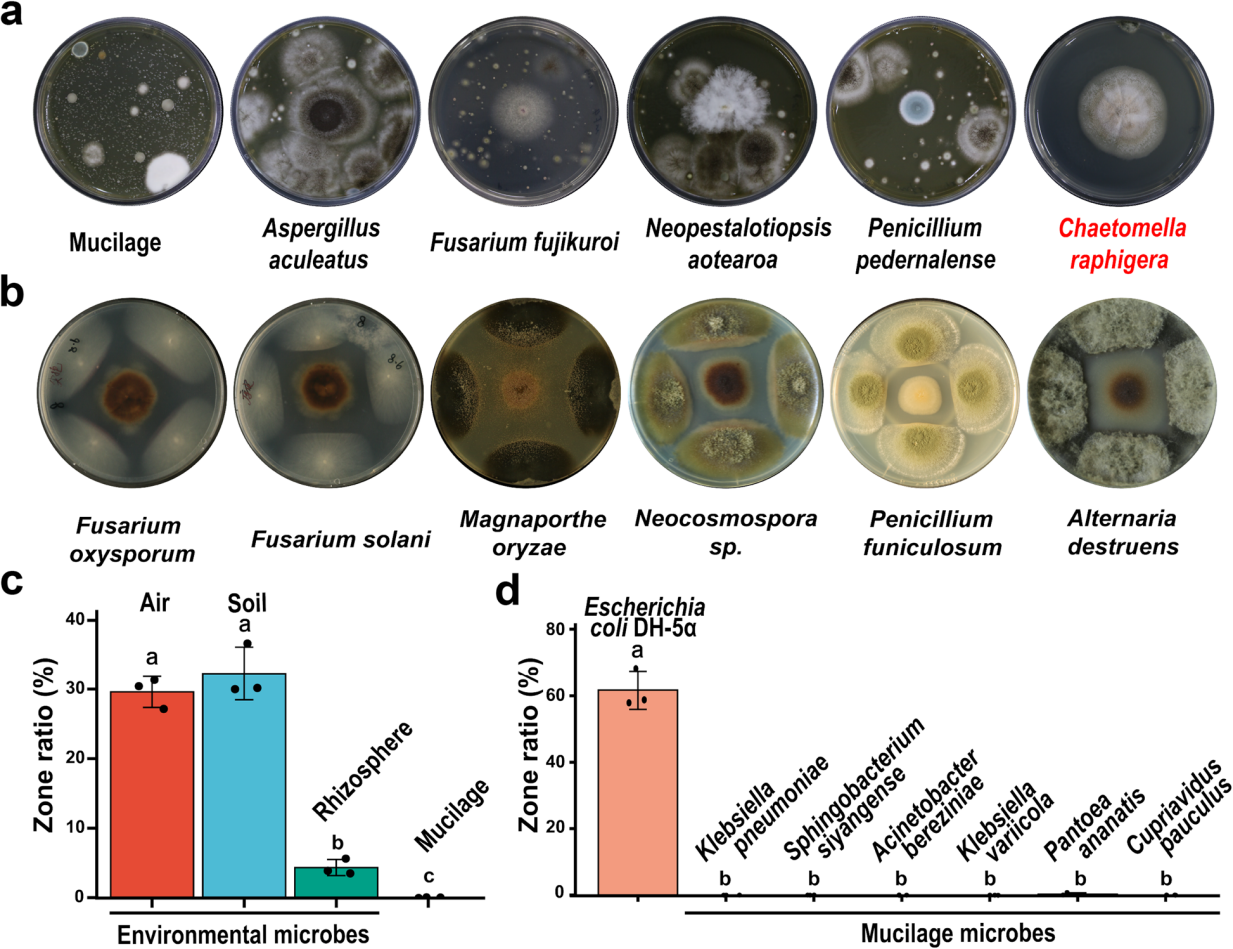
A candidate for friendly fungi in mucilage microhabitat and its defense against environmental microbes. Partners of mucilage and nitrogen-fixing bacteria: a broad-spectrum anti-microbe fungi (F-XTBG8). **a**, **b** Screening candidate in mucilage for resistance to environmental microbes in 1% and 10% PDA medium Only *C. raphigera* (F-XTBG8) shows resistance to the plant pathogenic fungi and various fungi from the environment (**b**). **c**,** d** By screening in the medium with added sterile mucilage (− 80 °C 14 d), F-XTBG8 fungal metabolites (right and red legend in bottom panel) are resistant to air and soil environmental bacteria and *Escherichia coli* DH-5α (**c**: air and soil), but not to mucilage diazotrophic bacteria (**c**: mucilage bacterial community strains, **d**: mucilage single microbe). Note: rhizosphere samples here refer to underground root samples that have just entered soil which converted from aerial roots. The zone ratio is equal to the zone of inhibition of the F-XTBG8 divided by the positive control (left, CK^+^: antibiotic streptomycin and tetracycline hydrochloride). Blank liquid medium was used as negative control (bottom, CK^−^). For the high-resolution photos of the microbial inhibition effects (Fig. c and d), referred to Supplementary Fig. 9

The alternative explanation for the homeostatic diazotrophic microbiota is that certain members of the community could selectively allow the growth of the diazotrophs, but inhibit unwelcomed environmental microbes. To identify potential friendly microbes in the mucilage microbiota, we collected 56 single-spore/single-colony bacterial and fungal isolates from mucilage samples and screened their broad-spectrum antibiotic activity by culturing on rich media exposed to open air. Through the 1% PDA medium with mucilage addition, we scanned that one fungal culture F-XTBG8 remained uncontaminated after 5 days of exposure to airborne microbes and was identified as *Chaetomella raphigera* by ITS sequencing (Figs. 5a, S8 and Table S10). Subsequent antibiotic tests demonstrated that this isolate of *C. raphigera* could inhibit the growth of over 100 common phytopathogens and environmental fungi including *Fusarium oxysporum, Fusarium solani,* and *Magnaporthe oryzae* (Figs. 5b and S8).

To identify the interaction between F-XTBG8 and other microorganisms, we further co-cultured F-XTBG8 and its metabolites with mucilage strains and other environmental microorganisms in the medium simulating mucilage microhabitat. Interestingly, the liquid culture of this *C. raphigera* strain demonstrated clear inhibitory effect against aerial and soil bacterial communities*,* but this effect significantly diminished when tested against rhizospheric bacteria of *H. rotundifolia*, and was completely abolished when tested against mucilage bacteria (Figs. 5c and S9). This may be because the root samples have just entered soil which converted from aerial roots and become normal underground roots. Consistently, liquid culture of *C. raphigera* suppressed the growth of a generic bacteria *Escherichia coli* DH-5α but showed no inhibitory effect against any of the six diazotrophic bacterial strains isolated from *H. rotundifolia* mucilage (Figs. 5d and S9). Two years of microbial amplicon sequencing of aerial root-mucilage samples, we found that this fungal genus was present in mucilage with a higher relative abundance than rhizosphere, and its sequencing reads in soil samples indicated that the fungus may be from planted soil or environment (Fig. S2d).

### Fungus *Chaetomella raphigera* genome and its antifungal effect

**Fig. 6:**
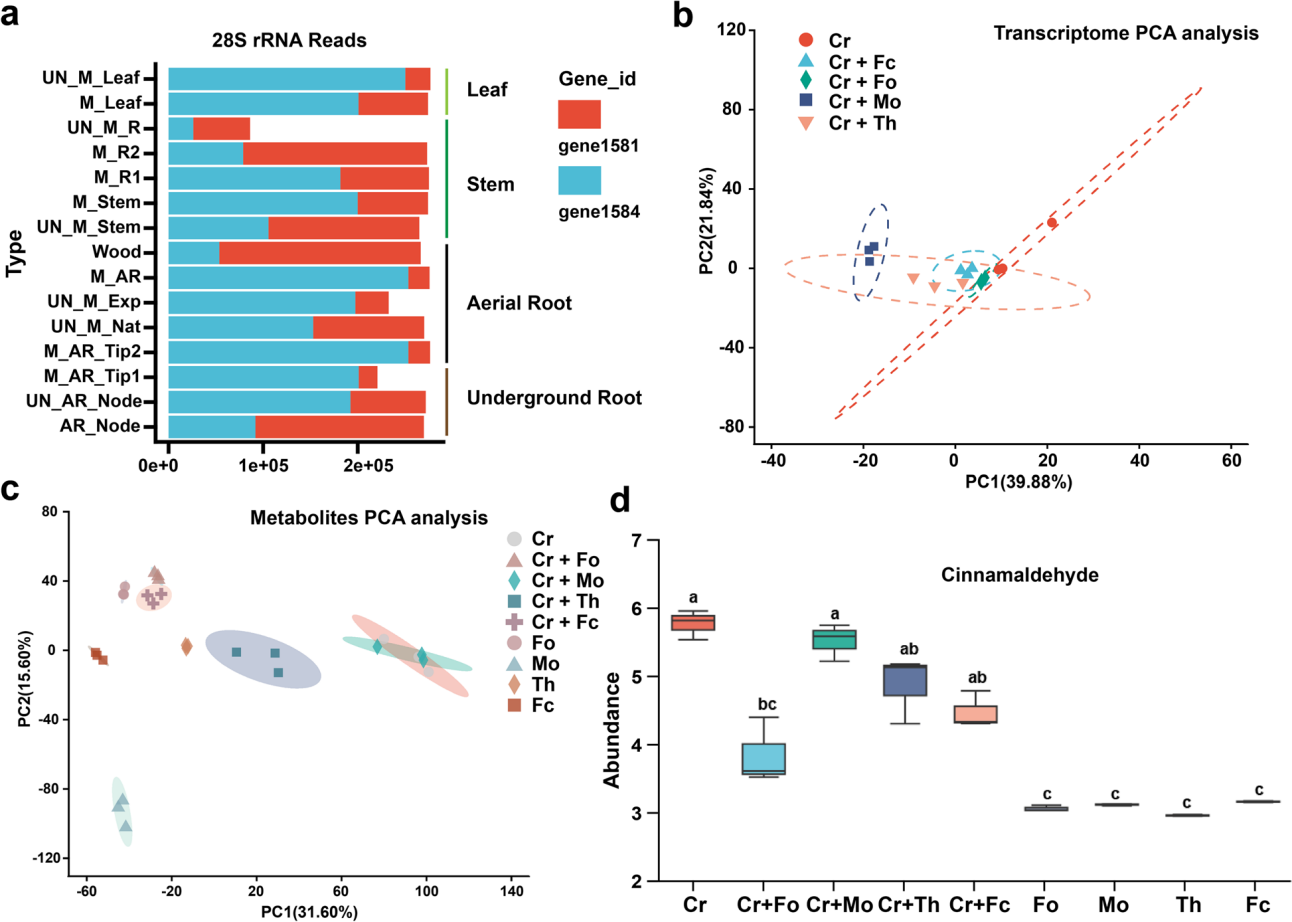
The genes of friendly fungi *C. raphigera* (F-XTBG8) were detected in plant tissues and its potential antifungal mechanisms. **a** Fungal 28 s rRNA genes are expressed in different plant tissues, indicating a friendly relationship with the host plant. AR (aerial root), R (underground root), M means mucilage, Tip (aerial root tip), UN_M means no aerial root mucilage, and UN_M means no aerial root. **b** Principal component analysis (PCA) plot based on the different transcriptome data. Genomic expression patterns for monocultivation of *C. raphigera* (Cr), and genomic expression patterns for cocultivation of *Fusarium concentricum* (Fc), *Magnaporthe oryzae* (Mo), *Fusarium oxysporum* (Fo), and *Trichoderma harzianum* (Th). Three biological replicates were analyzed for each group. **c** Principal component analysis (PCA) plot based on the different metabolites data. Metabolites analysis for monocultivation and cocultivation of *C. raphigera* (Cr) and four environmental fungi. **d** LC–MS analysis of monocultivation and cocultivation extracts (cinnamaldehyde) of *C. raphigera* (Cr) and four environmental fungi

To confirm that the friendly and “companion” fungi *C. raphigera* is selected by *H. rotundifolia* plant, we sequenced the monoculture fungus *C. raphigera* genome and the RNA-seq data of different 15 types of plant tissue (Figs. S10a and 6a). A total of 1710 of 8842 genes of the fungal genome were detected expression (reads FPKM > 10) in different plant tissues (Table S11). Interestingly, the fungal 28S rRNA genes (ribosomal gene 1581 and 1584) were widely found in different sample transcripts, which means that the fungi may habitat in leaves, stems, aerial roots, and underground roots of the host plant with or without aerial roots and mucilage (Fig. 6a). In addition, the fungal genes were found in the underground tissue and above part, implying that the fungi could be recruited from the from plant-borne (“vertical transmission”) or environment by the host (Table S11).

To further reveal the effect of how friendly fungi could inhibit a broad range of environmental fungi, the common pathogenic fungus *Fusarium* and *Magnaporthe* in agriculture were chosen for coculture, and transcription and metabolism analysis was performed (Fig. S8b). A total of *C. raphigera* 8080 transcripts (91.38% of genes) and 4723 metabolites were detected (Table S12). Moreover, in contrast to the monocultures of *C. raphigera* and pathogenic fungi, a broad accumulation of different metabolites were detected during cocultivation based on LC–MS analysis (Fig. 6c and Table S12), whereas there was no significant pathway difference for cocultivation transcriptome RNA-seq dates of *C. raphigera* (Fig. 6b–c and Table S12). Consistent with the previous medium coculture experiments, we found that friendly fungi and pathogenic fungi formed distinct inhibition zone and further hypothesized that the fungi may inhibit against other pathogens by releasing special metabolites. The transcriptome results of co-culture showed no widespread and coincident gene expression/pathway changes of *C. raphigera* could confirm this hypothesis. Statistical analysis showed that 51 metabolites significantly increased in coculture compared with those four pathogen monocultures (*P* < 0.01, Fig. S8b), and these metabolites not detected or very low content in four pathogen monocultures (Figs. 6d, S8 b-c and Table S12). Among them, 12 metabolites (subcluster 3) have been studied and shown that have a wide range of antifungal activities, such as cicerin, eugenol, 4-Isopropylbenzoic acid, and cinnamaldehyde [27, 28] (Figs. 6d and S8c). These results may demonstrate the widespread inhibit effect on environmental fungi by *C. raphigera* special metabolites.

## Discussion

Aerial roots are known to facilitate gas exchange in submergence-tolerant plants, provide additional mechanical support for climbing vines, or function as the primary site of nutrient uptake in marginal habitats [29]. Mucilage has been proposed to maintain a moisturized interface and provide lubrication and protection for aerial roots [30]. Microbiome studies in the last decade have revealed significant functions for the diverse microbial communities associated with the plant rhizosphere and have inspired similar investigation into the biochemical and microbial diversity in aerial root mucilage [3, 6, 7, 31, 32]. In a mucilage-secreting tropical maize landrace, microbes isolated from the carbohydrate-rich mucilage have been demonstrated to fix atmospheric nitrogen to promote plant growth [4].

In this study on *H. rotundifolia,* we confirmed that aerial root mucilage (ARM) and underground root exudate (URE) had distinct biochemical compositions from each other, both in terms of primary and specialized metabolites (Fig. 1). This biochemical differentiation correlated with the distinctive prokaryotic communities associated with aerial and underground roots (Fig. 2). Since the environmental microbial composition has a strong impact on the structure of plant-associated microbiota, the inherently different airborne and soilborne microbial communities is likely a predominant factor in deciding the microbial diversity in ARM and URE. Yet, the biochemical differences between ARM and URE likely play a role in structuring their associated microbiota as well. From the nutritional perspective, ARM was rich in amino acids, nucleotides, and carbohydrates, which provided a different niche from the lipid-rich URE (Fig. 1d) [5, 8, 33, 34]. Furthermore, the terpenoid and flavonoid-rich ARM could impose a distinct selective pressure on microbes compared to URE, which contained higher levels of alkaloids and phenolic acids (Fig. 1d).

Comparison of microbial community composition between URE and ARM revealed that several known diazotrophic bacterial genera were enriched in the ARM (Fig. 2c). In our study, based on bacterial function prediction and nitrogen-labeled experiment, we can conclude that the aerial root-mucilage microhabitat harbors diazotrophic microbiota such as diazotrophs *Klebsiella, Pantoea*, *Sphingobacterium*, *Herbaspirillum*, and *Burkholderia*. These bacteria are widely used model systems to study associative nitrogen fixation and are already well utilized for agricultural production [6, 22, 35–37]. Unfortunately, we were not able to produce any single-strain culture in these functional *Herbaspirillum* and *Burkholderia* genera under all accessible culturing conditions including anaerobic incubation and supplementation of sterilized *H. rotundifolia* mucilage. In the subsequent culture, hypoxia and selective culture of these microbes should be more simulated for mucilage microhabitat [6]. Consistently, the subsequent stable isotope tracking experiment showed that atmospheric nitrogen could be incorporated into plant chlorophyll, nitrogen, and biomass in an aerial root and mucilage-dependent manner (Fig. 3a–e). We further demonstrated that removal of aerial roots could significantly impact plant nitrogen content and biomass in a 4-month-long field experiment (Table. S5). In addition, based on the natural abundance of ^15^N, we found that the contribution of aerial root-mucilage system to %Ndfa was up to 54.85% (Fig. 3f). These results partially support the ecological relevance of ARM-mediated nitrogen fixation for plant fitness in situ, though the operation of aerial root removal could have complex influence on plant growth independent of the mucilage microbiota. In addition, aerial roots without mucilage also showed residual nitrogen fixation ability to some extent, which may be caused by the incompletely removed mucilage in aerial roots or endophytic nitrogen-fixing bacteria like the maize xylem [1].

Since nitrogen fixation involves dynamic nitrogen-carbon exchange between the symbiotic partners, we hypothesized that the aerial roots of *H. rotundifolia* needed to be highly active in nitrogen and carbon transportation. To test this hypothesis, we generated a chromosome-scale genome assembly of *H. rotundifolia* with PacBio CLR and Hi-C technologies and RNA sequencing data, which was then used for comparative transcriptomics analyses (Fig. 4). In support of our hypothesis, a number of sugar transporters were exclusively expressed in mucilage-bearing aerial roots when compared to aerial roots with no mucilage and underground roots (Fig. 4e). Interestingly, three of the five nitrogenous compound transporters highly expressed in underground roots were also expressed in aerial roots in a mucilage-dependent fashion (Fig. 4e) [38]. This expression pattern strongly suggests that the presence of ARM could induce the expression of nitrogenous compound transporters. This is consistent with previous observations in seagrass [2]. The nitrogen-fixing symbionts live within the seagrass root tissue, where it supplies amino acids and ammonia to the host in exchange for sugars [2]. In addition to transcriptional regulation that could maintain the diazotrophic mucilage microbiome, we hypothesize that this functional association may also be facilitated by adaptative evolution of the genome of *H. rotundifolia*. This potential genomic differentiation could be revealed by comparative genomics analysis of *H. rotundifolia* and sister species that cannot host nitrogen-fixing symbionts in the future.

The plant innate immune systems and specialized metabolites play important roles in shaping the host-associated microbiota [39–41]. Recent research has found that the host plant factors could shape the plant-association microbiota community by recruiting specific microbes while generating molecules that are toxic to others [8, 42]. Such function has been demonstrated for a large variety of plant special metabolites including triterpenes, coumarin, flavonoid, and benzoxazinoid are key compounds modulating plant microbiota composition [5, 43–48]. It is reasonable to speculate that the specialized compounds of aerial root mucilage recruit special microbiota as nutrients, in addition to some proteins and compounds that could serve as antibiotics to defend against pathogenic and environmental microbes or maintain the homeostasis of the mucilage-microbiota system. A hint at the answer to this question may be found in our potential mucilage and other compounds (Table S8): gallic acid, epigallocatechin gallate, phthalic anhydride, and others have a certain inhibitory effect on pathogens, but these compounds are low concentrations in mucilage and the relationship between metabolites and microbial homeostasis needs to be further explored. Whether plant special metabolites play an important regulatory role in the aerial root-mucilage microenvironment should further consider and expand list of candidate metabolites. In addition, preeminent researchers have shown that rice and maize roots secrete flavones that attract the enrichment of the rhizosphere diazotrophic bacteria [49], thereby nitrogen acquisition and promoting growth in nitrogen-poor soils [26]. This study inspires our research, we also found that aerial root mucilage has a higher content of flavonoids. It is still unknown how and which metabolites are recruited for shaping functional diazotrophic microbiota in the aerial root mucilage of *Heterotis rotundifolia* and worth further study with plant transcriptome (and metatranscriptomics), metabolomic, and metagenomics. In addition, we found that mucilage production always follows high humidity in the environment (Table S13), and future studies could reveal the causality between the dynamic basis of mucilage exudates and environmental factors.

The current hypothesis is that microbial homeostasis in plant roots is maintained by both microbiota-microbiota and host plant-microbiota and interactions, whereas little is known of those distinct outputs in maintaining microbial homeostasis between the plant and its root microbiota [13, 16]. Our discovery of *Chaetomella raphigera* (F-XTBG8)-the friendly fungi that helps mucilage and diazotrophic bacteria withstand pathogenic and environmental microbes-establishes the existence of such beneficial partnerships in aerial root-mucilage microhabitat. Previous study showed that the root microbiota homeostasis has an important regulatory role in nitrogen-fixing symbionts and the adaptation of plants to different environments. For instance, the rhizosphere microbiota *Bacillaceae* group promotes the nodulation of rhizobia *Sinorhizobium* and soybean growth under saline–alkali conditions [50]. Likewise, a recent study found that the facultative biotrophic fungus *Phomopsis liquidambaris* facilitates the migration of rhizobia from the soil to the peanut rhizosphere, thus triggering peanut-rhizobia nodulation [51]. Those relationships may be common in plant-microbiota interactions, and interesting findings may have fundamental practical implications [52]. This study for rhizosphere microecology implies that microbial interactions should also be considered when studying functional microbes. Search for “partner” or friendly microbes of plant growth-promoting bacteria and use synthetic communities (SynComs) for plant-microbial interaction studies and agro-ecosystem applications. Moreover, F-XTBG8 was not only nutritionally competitive with other environmental and pathogenic microbes, but its metabolites had inhibitory effects on those microbes and no inhibitory on diazotrophic bacteria in the mucilage. Interestingly, special antibacterial metabolites may produce by the friendly fungus F-XTBG 8 inhibiting the growth of other environmental and pathogenic microbes rather than diazotrophic bacteria. The mechanisms need to be further investigated, the recent study found that diazotrophic bacteria *Klebsiella* degrade various types of antibiotics and their genomes exhibit adaptations to toxins will help us further understand and further study friendly fungi and diazotrophic bacteria [53]. Further study could address microbial cross-feeding of friendly and functional microbes, which refers to interactions between microbes in which molecules metabolized by one microbe are further utilized by another microbe [54]. Extensive cultivation of these nitrogen-fixing microbes and further exploration of the molecular basis for the friendly coexistence of these microbes with friendly fungi from the genes, transporters, and metabolic gene clusters are necessary. Our study emphasized the key role the “friendly microbe” played in controlling the aerial root-mucilage-diazotrophic microbiota microenvironment and the biochemical conversation that dominates it. Further investigation of the fungal antimicrobial metabolites and broad-spectrum antimicrobe mechanisms of friendly fungi will help us to decipher the specific interaction of mucilage-microbiota and the underlying mechanism of microbial homeostasis. The predicted results of the metabolic genes of terpenoids and polyketides in its genome will help us to further target potential antibacterial compounds. Moreover, the origin of this fungus and diazotrophic bacteria needs to be further determined which may be from plant-borne (“vertical transmission”) or environment unresolved. Collectively, our concepts provide a study paradigm for understanding how plants may engage with functional microbiota while restricting pathogens and environmental microbes.

**Fig. 7:**
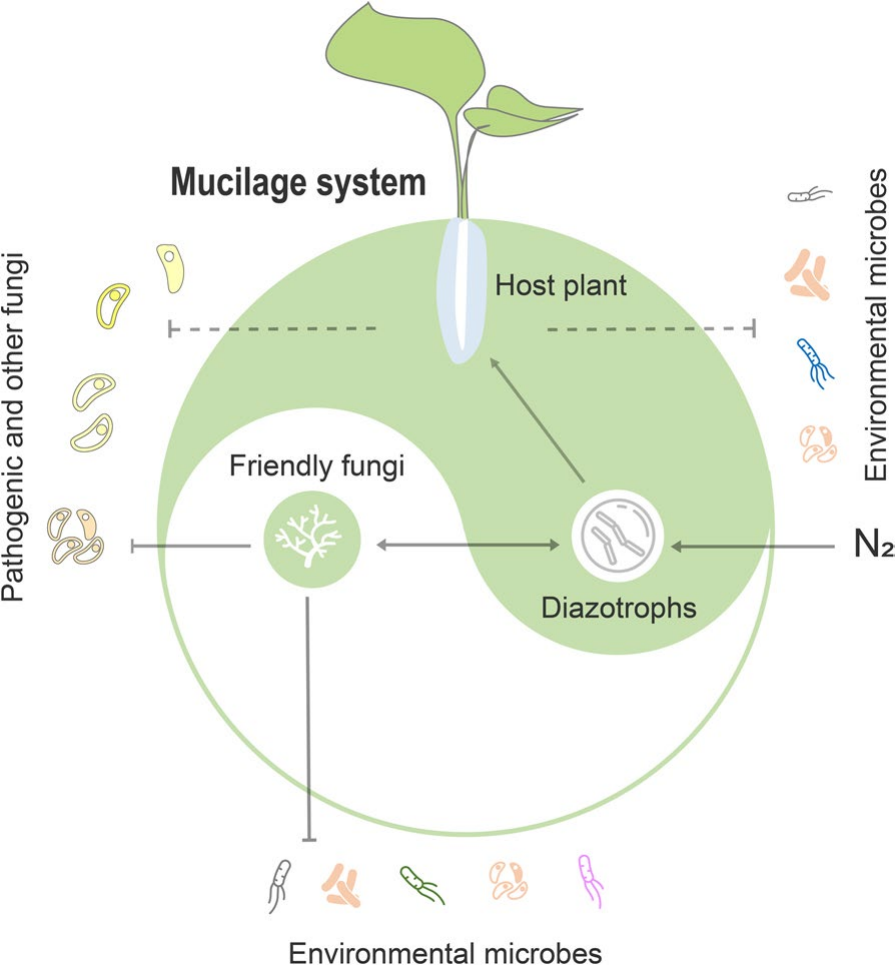
The model of aerial root-mucilage-microbiota system. The friendly relationship between friend fungi and diazotrophic microbiota in the aerial root-mucilage microhabitat. The friend fungi have a broad spectrum of resistance to environmental and pathogenic microbes rather than functional bacteria. The aerial root-mucilage may screen and resist microbes of environment

In summary, our study found a novel role for aerial root-mucilage microhabitat in nitrogen uptake, and extended the concept of rhizosphere, that aerial roots can still perform the same biological functions as underground roots. More importantly, the discovery of friendly fungi in mucilage further advances our understanding of the homeostasis mechanisms of specific functional microbiota in the microenvironment (Fig. 7). This further confirms that plants actively mediate plant-microbiota interactions and maintain microbial homeostasis and could have an important impact on nitrogen use efficiency, rapid growth, and invasion of *H. rotundifolia*. The friendly microbe insight provides important understanding for diverse problems concerning plant microbiota assembly and environmental microbes. We hope that the aerial root-mucilage-functional microbiota system established in this study will enable basic biological insights to be gained for biological interactions.

## Methods

### Plant samples

*H. rotundifolia* maintained by Xishuangbanna Tropical Botanical Garden (Xishuangbanna, China) was sampled between June 2019 and December 2021. The aerial roots have been removed in the wild and greenhouse experiment in Xishuangbanna Tropical Botanical Garden. A string is used as a barrier to keep the aerial roots entering the ground or soil during the growth of the plant. After 4 months of the experiment, the stem length was recorded, and the dry weight and nitrogen content were analyzed after drying.

### Metabolite profiling

#### Aerial root mucilage and underground root exudate collection

In May 2020, we chose the creeping *H. rotundifolia* plants that were mature enough to have roots at various stages to sample the aerial root mucilage and underground root exudate. Sterile forceps were used to load the aerial root mucilage into a 50-mL centrifuge tube. For underground root exudate collection, roots were repeatedly washed and shaken in a 200-mL deionized water and collected promptly after root washing for 2 h by 60 rpm. All mucilage and exudate samples were immediately frozen to − 80 °C and then freeze-dried (MM400, Retsch) for 1.5 min at 30 Hz. After mixing, take 40 mL sample into 50 mL centrifuge tube and then immerse the sample in liquid nitrogen. Powder (100 mg) was weighed and extracted overnight at 4 °C with 1 mL 70% methanol, followed by centrifugation for 10 min at 12 000 g. The supernatants were collected separately and combined, followed by filtration of 0.22-mm pore size. For metabolome analysis, mucilage and exudate samples were analyzed using ultrahigh-performance liquid chromatography (SHIMADZU Nexera X2)-tandem mass spectroscopy (Applied Biosystems 4500 QTRAP) (UPLC-MS/MS) based widely targeted metabolome method by Wuhan Metware Biotechnology Co., Ltd. (Wuhan, China) (http://www.metware.cn/). Carbohydrate contents were detected based on an Agilent 7890B gas chromatograph coupled to a 7000D mass spectrometer (GC–MS) platform by Metware. Concentration determination of candidate compounds (gallic acid, epigallocatechin gallate, phthalic anhydride, and others) in mucilage using high-performance liquid chromatography and ultra-performance liquid chromatography-mass spectrometry (Supplementary Methods 1 for details regarding the protocol).

#### Mucilage and soil DNA extraction, 16S rRNA, and ITS gene sequencing

In our study, the rhizosphere soil was defined and collected as previous research [4]. Aerial root mucilage and underground rhizosphere soil and bulk soil sample DNA were extracted using the FastDNA SPIN Kit for Soil (MP Biomedicals, USA) according to the manufacturer’s protocols. The fungal ITS1 region (ITS1F/ITS2R) [55] and the bacterial 16S rRNA gene (V3-V4, 338F/806R) [56] were amplified. Amplicon libraries were sequenced on the Illumina MiSeq PE300 platform by the Majorbio Bio-Pharm Technology Co., Ltd. (Shanghai, China).

The 16S rRNA and ITS gene sequencing reads were demultiplexed by Fastp (version 0.20.0) [57] and merged with FLASH (version 1.2.7) [58] following: operational taxonomic units (OTUs) with 97% similarity were clustered using UPARSE (version 7.1) [59]. Taxonomic assignment was performed using the bacterial SILVA reference database (v12_8) and fungal UNITE database (v7.0). Functional prediction (Tax4Fun, PICRUst1/2, FAPROTAX, BugBase phenotype prediction) was analyzed on the free online platform of the Majorbio Cloud Platform (www.majorbio.com) [1].

#### Bacteria and fungi isolation in mucilage and rhizosphere

Fungi isolated from the mucilage and rhizosphere were placed onto potato dextrose agar (PDA, Hopebio, Qingdao) and incubated for 7 days (in the dark at 28 °C). Five grams of rhizospheric soil was weighed into a 50-mL centrifuge tube, and 10^–3^ and 10^–4^ diluents were soaked up with diluted spreading onto tryptic soy agar (TSA, Hopebio, Qingdao), LB agar, and nitrogen-free agar (Ashby, Rhizobium, Associations and Nitrogen-free Culture-medium, Hopebio, Qingdao) to isolate diazotrophic bacteria. To simulate the native habitats to increase the diversity of cultivatable microbes, we attempted to add sterilized ARM of *H. rotundifolia* into commercial media (final concentration 10^–4^ diluents) and incubated the culture under both aerobic and anaerobic conditions. Pure culture isolates were obtained by plate-streaking or single-spore isolation. ITS and 16S rRNA genes of the isolated strains were amplified with fungal primers ITS1F (5′-CTTGGTCATTTAGAGGAAGTAA-3′) and ITS4R (5′-TCCTCCGCTTATTGATATGC-3′) and bacterial universal 27F (5′-GAGAGTTTGATCCTGGCTCAG-3′) and 1492R (5′-ACGGATACCTTGTTACGACT-3′). ITS and 16S sequences were aligned with the NCBI ITS (fungi) and 16S rRNA sequences (bacteria) databases by Nucleotide BLAST (https://blast.ncbi.nlm.nih.gov/Blast.cgi) to determine the approximate phylogenetic affiliation. The core nitrogen fixation genes (*nif*) from known diazotrophs and maize mucilage microbiota as previously published [4, 60] as a reference.

#### ^15^N_2_ gas-enrichment experiments and pheophytin analysis

The three types of no-aerial root, no-mucilage, and aerial root-mucilage plants (none of them have underground roots, about 18 cm in length) were collected from the healthy *H. rotundifolia* plant. For the Isotope ratio mass spectrometry (IRMS) study, we constructed an experimental device for ^15^N_2_-labeling experiments, a clear 565 mL plastic bottle, equipped with an intake valve and a device for fixing plants (Fig. 2a). After simply washing the sampled plant material with sterile water, place the sterile filter paper on the bottom layer of bottle and add a 2-mL sterile water for moisturizing. Then, the 0% (negative controls), 5%, 10%, and 20% (v/v) of ^15^N_2_ gas (99.9% purity, Wuhan Isotope Technology, Co., Ltd., Wuhan, China) was pumped into the bottle, and the samples were moved to phytotron at 25 °C for 72 h.

For the nitrogen-fixation bacteria strain, the bacteria liquid of logarithmic growth stage (2 weeks of age) was added into 125 mL conical flask, sealed, and replaced with 99.9% ^15^N_2_ by 20% of the volume of gas in the flask with a syringe. The culture was continued at 28 °C for 72 h, and strain cells were collected through Whatman glass microfiber filter (GF/F, GE Healthcare). After drying, grinding and weighing, the ^15^N of individual aerial root, young and old leaf, stem from each plant and bacterial strains were measured using EA-HT elemental analyzer (Thermo Fisher Scientific, Inc., Bremen, Germany) coupled to an isotope ratio mass spectrometer (DELTA V Advantage, Thermo Finnigan). The isotopic composition analysis was performed at Tsinghua university Stable Isotope Facility (Shenzhen, China) and Central laboratory of Xishuangbanna Tropical Botanical Garden (Xishuangbanna, China).

For the pheophytin analysis, chlorophyll of leaf and stem were extracted, similarly to previously reported methods [4, 61, 62]. ^14^N_2_-treated plants with mucilage and non-mucilage were used as negative controls [62]. Chlorophyll was converted to pheophytin by acid treatment for obtaining pheophytin, following as described Pheophytin isotope abundances (m·z^−1^: 871.57–875.57) were analyzed by quantitative time-of-flight (qTOF) mass spectrometry on the liquid chromatograph-mass spectrometer (LC–MS, Agilent 6545). The analysis was similar to previously reported methods [62].

### ^15^N natural abundance

The biological nitrogen fixation sources (%Ndfa) proportion was analyzed using the ^15^N natural abundance (δ, ‰) of the mucilage-producing-fixing plant (δ^15^N, fixing plant-ARM) and non-aerial root-reference plants (δ^15^N, reference plants). In 2020 and 2021, nearly 12 individual mucilage-producing samples and eight non-aerial root-reference plant samples were analyzed. The percentage of nitrogen derived from nitrogen fixation (%Ndfa) was calculated as follows:

$$\%\mathrm{Ndfa}\;=\left(\mathrm\delta^{15}{\mathrm N}_{\mathrm{reference}}\;-\;\mathrm\delta^{15}{\mathrm N}_{\mathrm{fixing}\;\mathrm{plant}-\mathrm{ARM}}\right)/\left(\mathrm\delta^{15}{\mathrm N}_{\mathrm{reference}}\;-\;\mathrm B\right)\times100\%$$

The “δ^15^N” is stable nitrogen isotopes, “ref” is the value from non-aerial root-reference plants, “fixing plant-ARM” is the mucilage-producing-fixing plant, and “B” is the ^15^N abundance in the air, assumed to be 0.0‰.

#### Microbial antagonism experiment

To identify mucilage compounds and microbes with broad-spectrum resistance, the compounds and isolates were purified and transferred to a new medium plate for 24 h in advance (bacteria 12 h) and then placed outdoors (exposed to airborne microbial infection) 5 days with the cover of the plate open. Medium plate that did not grow other microbes were considered to have broad-spectrum resistance. To simulate the mucilage environment, plates consist of sterile mucilage and agar. For mucilage sterilization, it was placed in − 80 °C refrigerator for 14 days, melted at room temperature, and then added to the high-temperature sterilized agar. The negative control was melted mucilage cultured in PDA and TSA medium. The efficacy of fungi strain “F-XTBG8” was tested against environment and pathogenic fungi on potato dextrose agar (PDA) medium plates. Fungal culture plate agar discs (6 mm) of environment and pathogenic fungi were disposed in all around at the center of F-XTBG8 strain in a square at 1.8 cm distance and incubated at 30 °C until mycelial growth had filled medium plates. The Oxford cup method was used for the mucilage compounds, fungal metabolite, and bacteria antagonism experiment, the cup height of 10 mm, inner diameter of 6 mm, and outer diameter of 8 mm. Then, 100 uL of each bacterial suspension (OD_600_ = 1) was spread evenly on plates (mucilage compounds: 1% PDA and 1% TSA, fungal metabolites: sterile mucilage with agar), while three sterilized Oxford cups were placed in each plate, and three cups were inoculated separately with 200 uL of antibacterial streptomycin and tetracycline hydrochloride (mix, positive controls, CK^+^), PDB of fungal metabolite (F-XTBG8), and bulk PDB (negative control, CK^+^). The plates of inoculated bacterial suspension were cultured at 30 °C for 12 h. The antagonistic zones (the bacterial growth inhibition zone minus cup outer diameter) were measured to evaluate the antibacterial effects of F-XTBG8 on different bacteria.

#### H. rotundifolia genome sequencing, assembly, and annotation

Genomic DNA was extracted using the QIAGEN DNaesy Plant Mini Kit according to the manufacturer’s protocols. The extracted DNA molecules were sequenced by PacBio Sequel (Pacific Biosciences of California, Menlo Park, CA, USA) platforms. The CLR reads were assembled using mecat2 (20,190,226) with default parameters. Draft genome was corrected by arrow and pilon. Polished contigs were anchored to chromosome by Hi-C reads. First, Hi-C reads were mapped to the polished *H. rotundifolia* genome using BWA (bwa-0.7.17) and Lachesis with default parameters [63]. Paired reads with mate mapped to a different contig were used to do the Hi-C-associated scaffolding. Lachesis was further applied to cluster, order, and orient the clustered contigs. Two methods were combined to identify the repeat contents in *H. rotundifolia* genome, homology-based, and de novo prediction. The repeat contents found by these two methods were merged by repeat masker. Protein-coding genes of the *H. rotundifolia* genome were predicted by three methods, including ab initio gene prediction, homology-based gene prediction, and RNA-Seq-aided gene prediction (Supplementary Methods 2 for genome sequencing, assembly, and annotation details).

#### H. rotundifolia RNA isolation and transcriptome analysis

RNA samples were extracted using the Trizol reagent following the manufacturer’s recommendations (Invitrogen, CA, USA) and then sequenced using the MGI-SEQ 2000 platform (Supplementary Methods 2 for library construction and sequencing details). Low-quality reads were filtered out by SOAPnuke software [64], and clean reads were mapped to *H. rotundifolia* genome using bowtie2 software [65]. Gene expression levels were estimated using FPKM values (fragments per kilobase per million fragments mapped) by the RSEM software [66]. DESeq2 [67] was used to evaluate differential expression genes between different root samples. Genes with fold change > 1 or ≤ 1, and FDR < 0.05 were differentially expressed genes.

### Statistical analysis and data normalization

All data analysis was conducted in R and visualized using the ggplot2 and igraph packages. The corrected *P* values were used as the significance threshold for differentially expressed genes. The microbial alpha diversity, including the Shannon, Chao1, and Simpson indices, was determined using Mothur v. 1.34.4. The PCoA was performed to examine the similarities and dissimilarities within the different groups. Some analysis and function prediction such as Spearman’s correlations, PICRUSt, FUNGuild, and others were analyzed on the free online platform of the Majorbio Cloud Platform (www.majorbio.com). The base R package “stats” (v. 3.4.1) was used to perform the two-tailed Wilcoxon rank-sum test (wilcox.test function). ANOVA and mean separation using least significant difference (*P* = 0.05) for each location. T test and two-way ANOVA test were significant at *P* = 0.05.

## Supplementary Information

**Additional file 1: Figure S1.** Aerial root mucilage (ARM) and underground root exudate (URE) compound of *H. rotundifolia*. **Figure S2.** Fungal diversity and community of aerial root mucilage (mucilage) and underground rhizosphere soil (rhizosphere). **Figure S3.** Differentiate analysis, function and phenotypic prediction of mucilage and rhizosphere bacteria. **Figure S4.** Cultured bacteria and their nitrogen-fixing capacity. **Figure S5.** Cultured bacteria and their nitrogen-fixing capacity. **Figure S6.** Estimated genome size of *H. rotundifolia* by flow cytometry. **Figure S7.** Resistance of mucilage compound to environmental microbes. **Figure S8.** Resistance of F-XTBG8 to pathogenic and environmental fungi. **Figure S9.** A candidate in mucilage microhabitat and its defense against environmental microbes but not mucilage bacteria. **Figure S10.** Genome, transcriptome and metabolome analysis of *C. raphigera* (Cr).

**Additional file 2: Table S1.** Relative content of different compounds and carbohydrates of aerial root mucilage (ARM) and underground root exudate (URE) **Table S2.** Fungal and bacterial OTU of different samples. **Table S3.** Differentially bacterial and fungal genera of rhizosphere and mucilage. **Table S4.** Cultured bacteria and their nitrogen-fixing capacity. **Table S5.** Plant nitrogen enrichment, natural abundance, nitrogencontent, length and dry weight analysis. **Table S6.** Results of plant genome sequencing, assembly, and annotation. **Table S7.** GO and KEGG enrichment of differential sample. **Table S8.** Candidates for follow-up experiments in mucilage compounds. **Table S9.** The mucilage compounds for anti-microbe activity test. **Table S10.** Antagonism of F-XTBG8 against different fungi and bacteria.  **Table S11.** Fungal genome was detected and expressed (reads FPKM>10) in different tissues of the plant. **Table S12.** Different metabolites analysis for monocultivation of *C. raphigera *(Cr) and cocultivation with environmental fungi. **Table S13.** Record of mucilage production and meteorological factors.

**Additional file 3: Method S1.** Carbohydrate and widely targeted metabolites profiling.** Method S2.** Plant Genome and transcriptome analysis.

## Data Availability

The genome, transcriptome, microbiome, and other related data reported in this paper have been deposited in the National Genomics Data Center (NGDC), under accession code BioProject: PRJCA009607 (GSAs: CRA005765, Samples: SAMC778622, GWH: WGS025680, https://ngdc.cncb.ac.cn/).
