# Supplementary material for: Microbiota-mediated nitrogen fixation and microhabitat homeostasis in aerial root-mucilage
Source: Microbiome. 2023 Apr 21;11:85. doi: 10.1186/s40168-023-01525-x (PMC10120241; doi:10.1186/s40168-023-01525-x)
Supplement: Supplementary file 2 — Additional file 1: Figure S1. Aerial root mucilage (ARM) and underground root exudate (URE) compound of H. rotundifolia. Figure S2. Fungal diversity and community of aerial root mucilage (mucilage) and underground rhizosphere soil (rhizosphere). Figure S3. Differentiate analysis, function and phenotypic prediction of mucilage and rhizosphere bacteria. Figure S4. Cultured bacteria and their nitrogen-fixing capacity. Figure S5. Cultured bacteria and their nitrogen-fixing capacity. Figure S6. Estimated genome size of H. rotundifolia by flow cytometry. Figure S7. Resistance of mucilage compound to environmental microbes. Figure S8. Resistance of F-XTBG8 to pathogenic and environmental fungi. Figure S9. A candidate in mucilage microhabitat and its defense against environmental microbes but not mucilage bacteria. Figure S10. Genome, transcriptome and metabolome analysis of C. raphigera (Cr). [file 40168_2023_1525_MOESM1_ESM.zip › Fig. S1-10.pptx]

## Slide 1
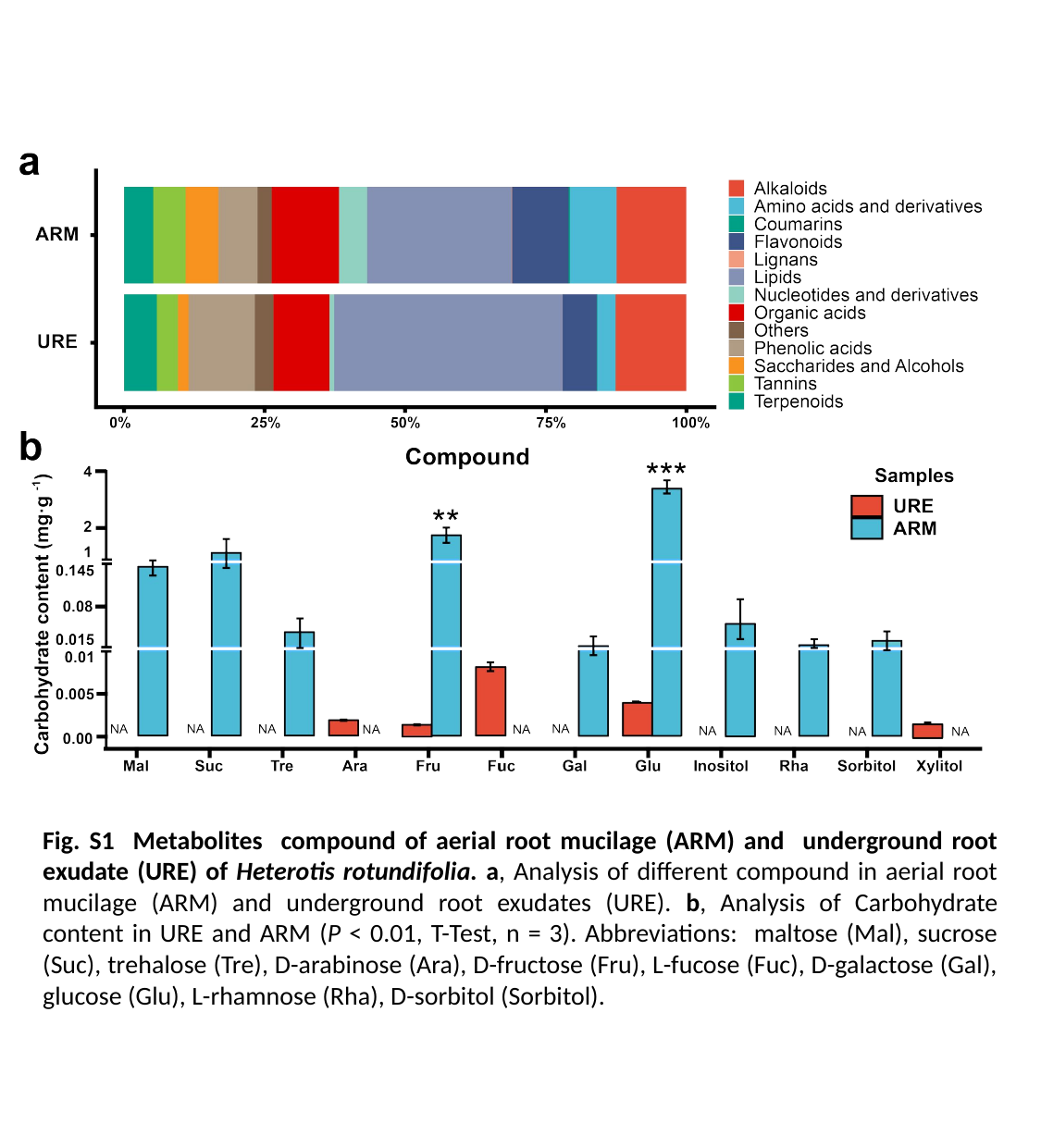

Fig. S1 Metabolites compound of aerial root mucilage (ARM) and underground root exudate (URE) of Heterotis rotundifolia. a, Analysis of different compound in aerial root mucilage (ARM) and underground root exudates (URE). b, Analysis of Carbohydrate content in URE and ARM (P < 0.01, T-Test, n = 3). Abbreviations: maltose (Mal), sucrose (Suc), trehalose (Tre), D-arabinose (Ara), D-fructose (Fru), L-fucose (Fuc), D-galactose (Gal), glucose (Glu), L-rhamnose (Rha), D-sorbitol (Sorbitol).

## Slide 2
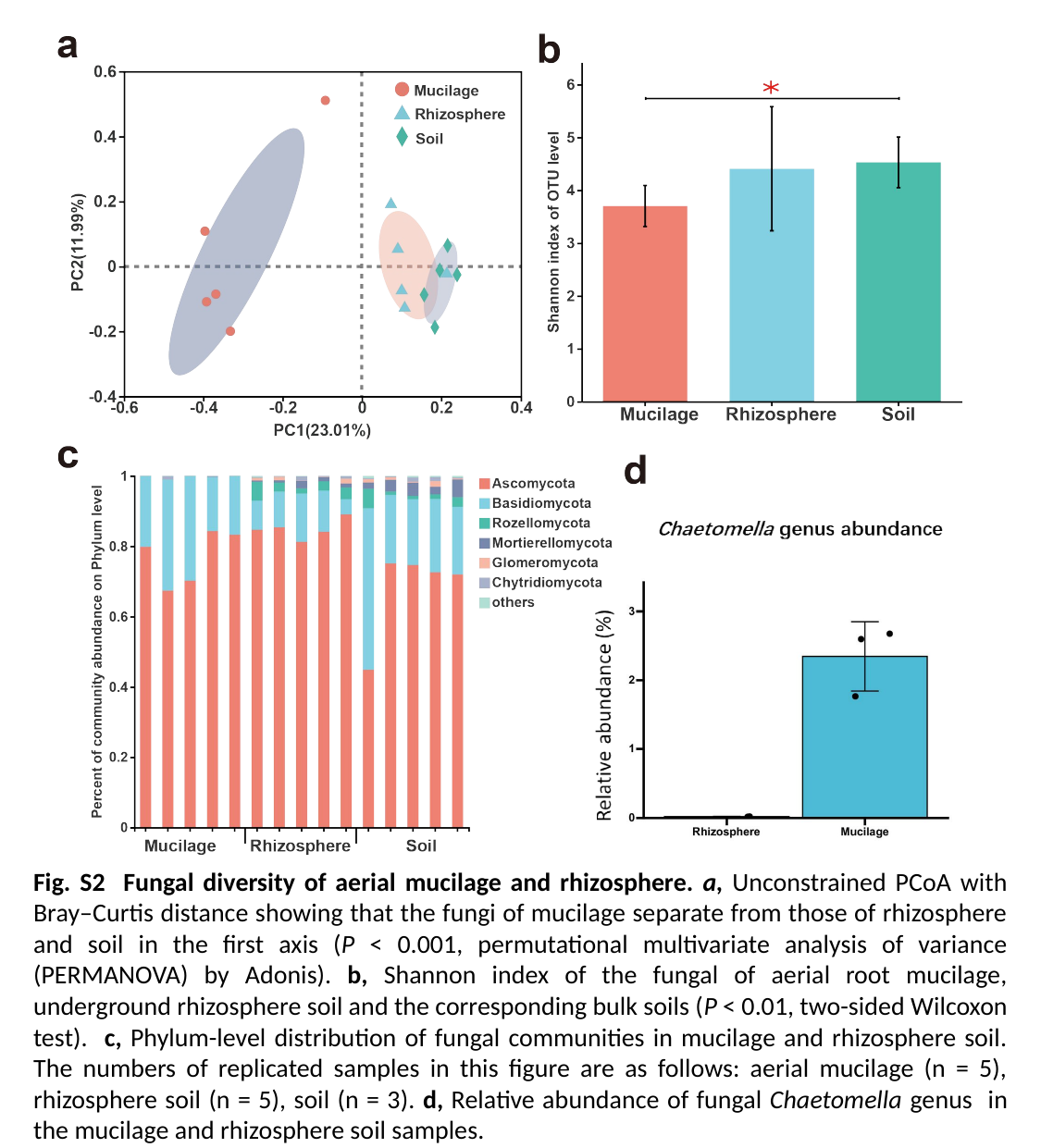

Fig. S2 Fungal diversity of aerial mucilage and rhizosphere. a, Unconstrained PCoA with Bray–Curtis distance showing that the fungi of mucilage separate from those of rhizosphere and soil in the first axis (P < 0.001, permutational multivariate analysis of variance (PERMANOVA) by Adonis). b, Shannon index of the fungal of aerial root mucilage, underground rhizosphere soil and the corresponding bulk soils (P < 0.01, two-sided Wilcoxon test). c, Phylum-level distribution of fungal communities in mucilage and rhizosphere soil. The numbers of replicated samples in this figure are as follows: aerial mucilage (n = 5), rhizosphere soil (n = 5), soil (n = 3). d, Relative abundance of fungal Chaetomella genus in the mucilage and rhizosphere soil samples.

## Slide 3
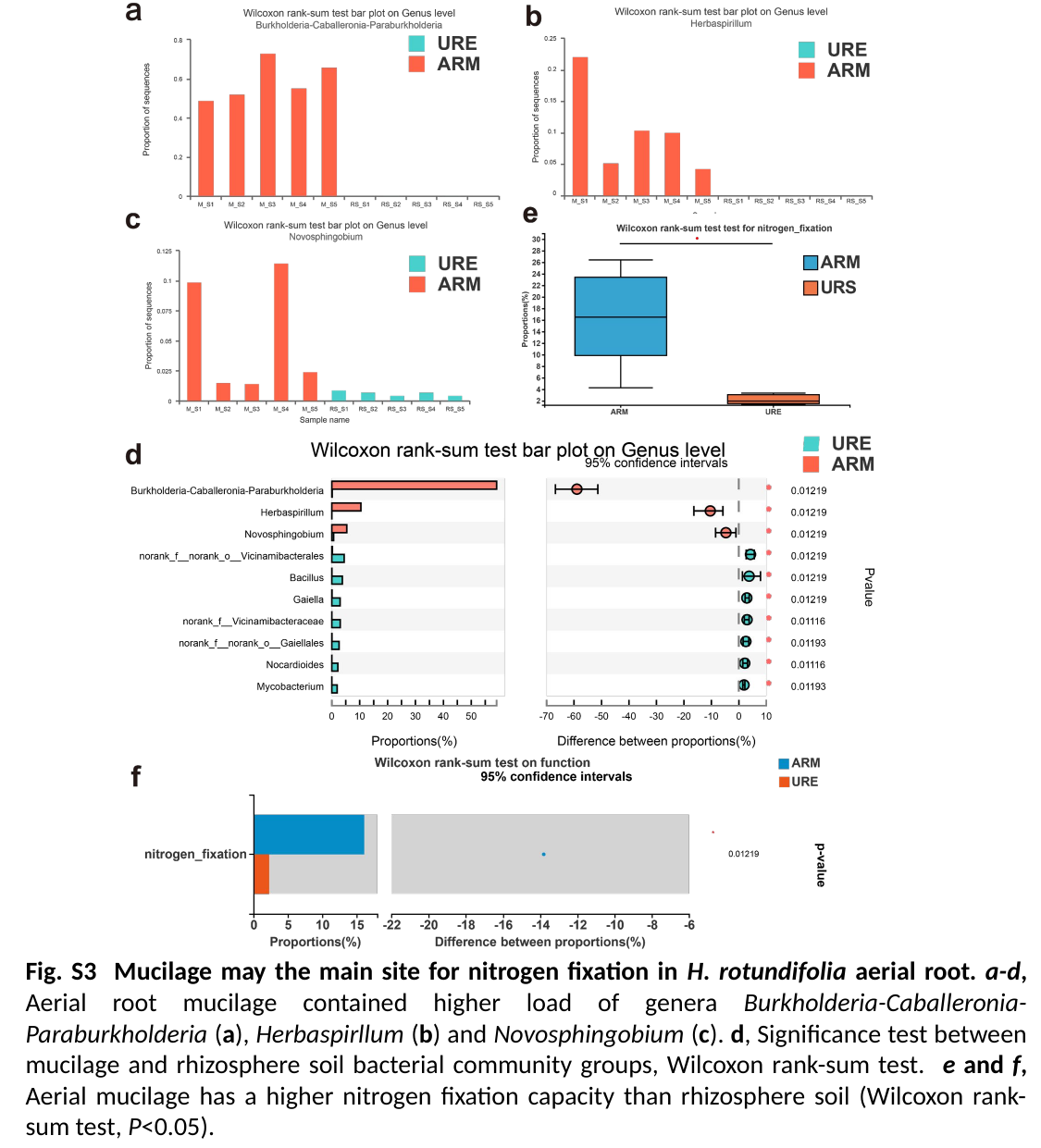

Fig. S3 Mucilage may the main site for nitrogen fixation in H. rotundifolia aerial root. a-d, Aerial root mucilage contained higher load of genera Burkholderia-Caballeronia-Paraburkholderia (a), Herbaspirllum (b) and Novosphingobium (c). d, Significance test between mucilage and rhizosphere soil bacterial community groups, Wilcoxon rank-sum test. e and f, Aerial mucilage has a higher nitrogen fixation capacity than rhizosphere soil (Wilcoxon rank-sum test, P<0.05).

## Slide 4
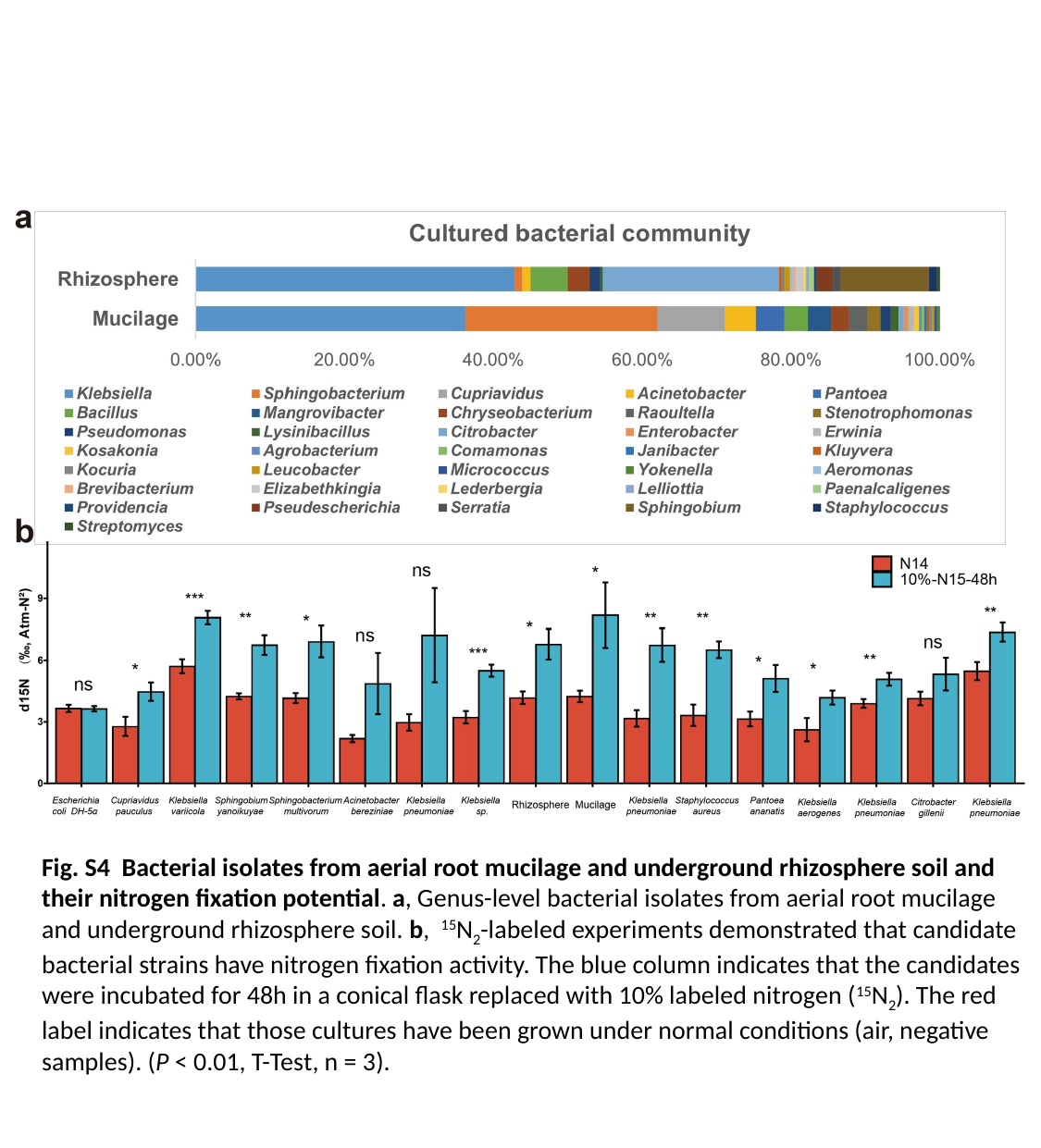

Fig. S4 Bacterial isolates from aerial root mucilage and underground rhizosphere soil and their nitrogen fixation potential. a, Genus-level bacterial isolates from aerial root mucilage and underground rhizosphere soil. b, 15N2-labeled experiments demonstrated that candidate bacterial strains have nitrogen fixation activity. The blue column indicates that the candidates were incubated for 48h in a conical flask replaced with 10% labeled nitrogen (15N2). The red label indicates that those cultures have been grown under normal conditions (air, negative samples). (P < 0.01, T-Test, n = 3).

## Slide 5
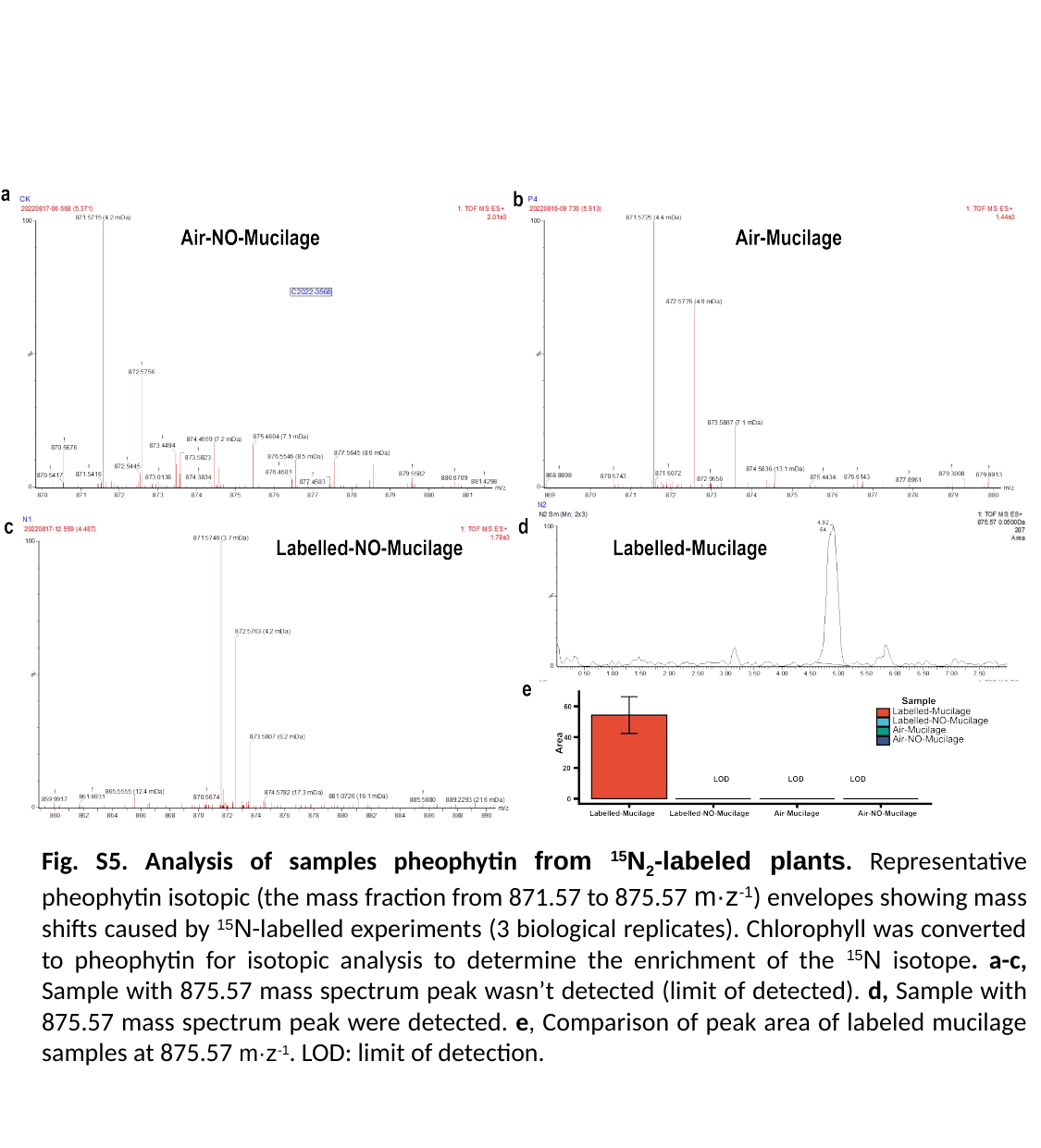

Fig. S5. Analysis of samples pheophytin from 15N2-labeled plants. Representative pheophytin isotopic (the mass fraction from 871.57 to 875.57 m·z-1) envelopes showing mass shifts caused by 15N-labelled experiments (3 biological replicates). Chlorophyll was converted to pheophytin for isotopic analysis to determine the enrichment of the 15N isotope. a-c, Sample with 875.57 mass spectrum peak wasn’t detected (limit of detected). d, Sample with 875.57 mass spectrum peak were detected. e, Comparison of peak area of labeled mucilage samples at 875.57 m·z-1. LOD: limit of detection.

## Slide 6
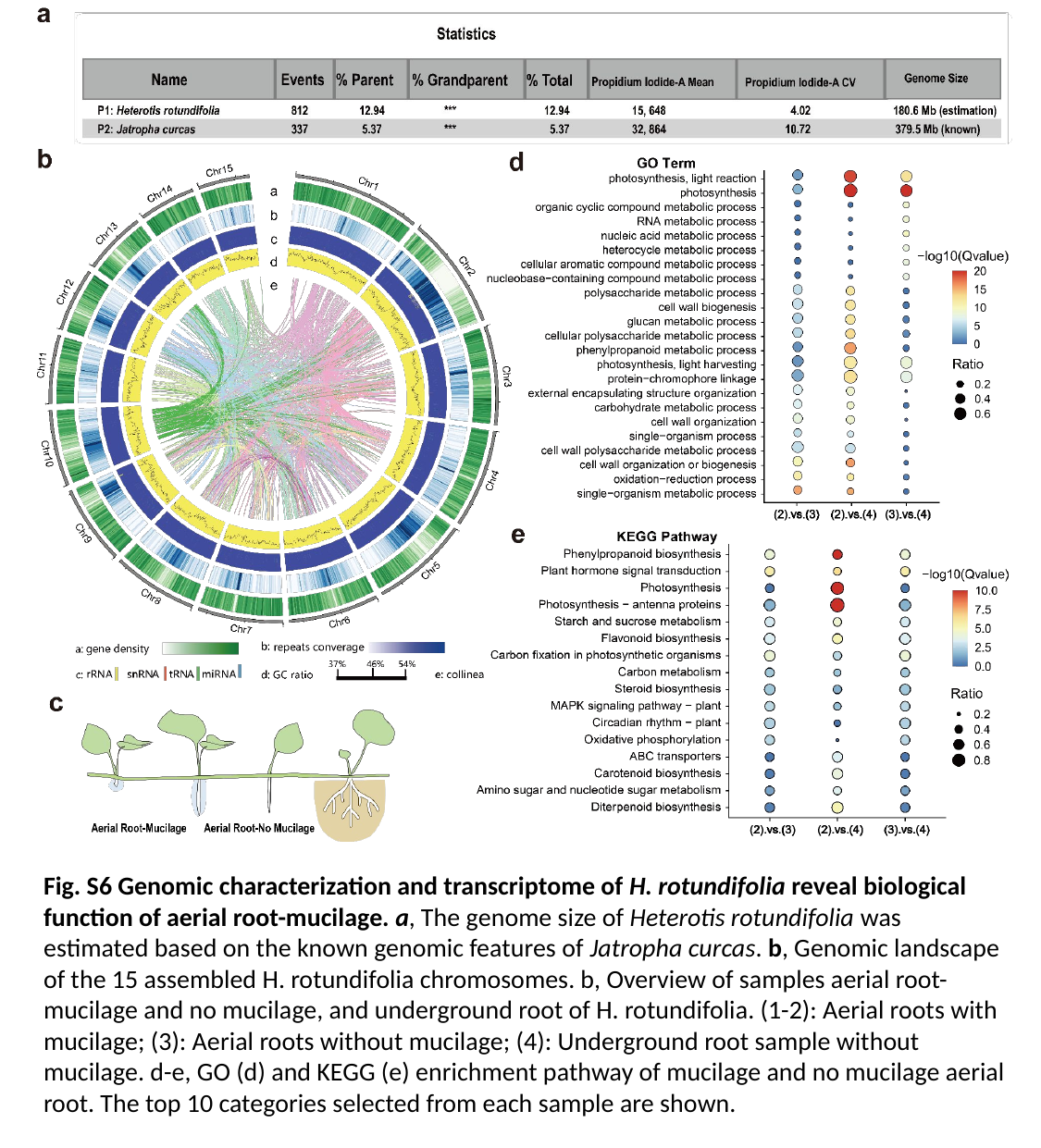

Fig. S6 Genomic characterization and transcriptome of H. rotundifolia reveal biological function of aerial root-mucilage. a, The genome size of Heterotis rotundifolia was estimated based on the known genomic features of Jatropha curcas. b, Genomic landscape of the 15 assembled H. rotundifolia chromosomes. b, Overview of samples aerial root-mucilage and no mucilage, and underground root of H. rotundifolia. (1-2): Aerial roots with mucilage; (3): Aerial roots without mucilage; (4): Underground root sample without mucilage. d-e, GO (d) and KEGG (e) enrichment pathway of mucilage and no mucilage aerial root. The top 10 categories selected from each sample are shown.

## Slide 7
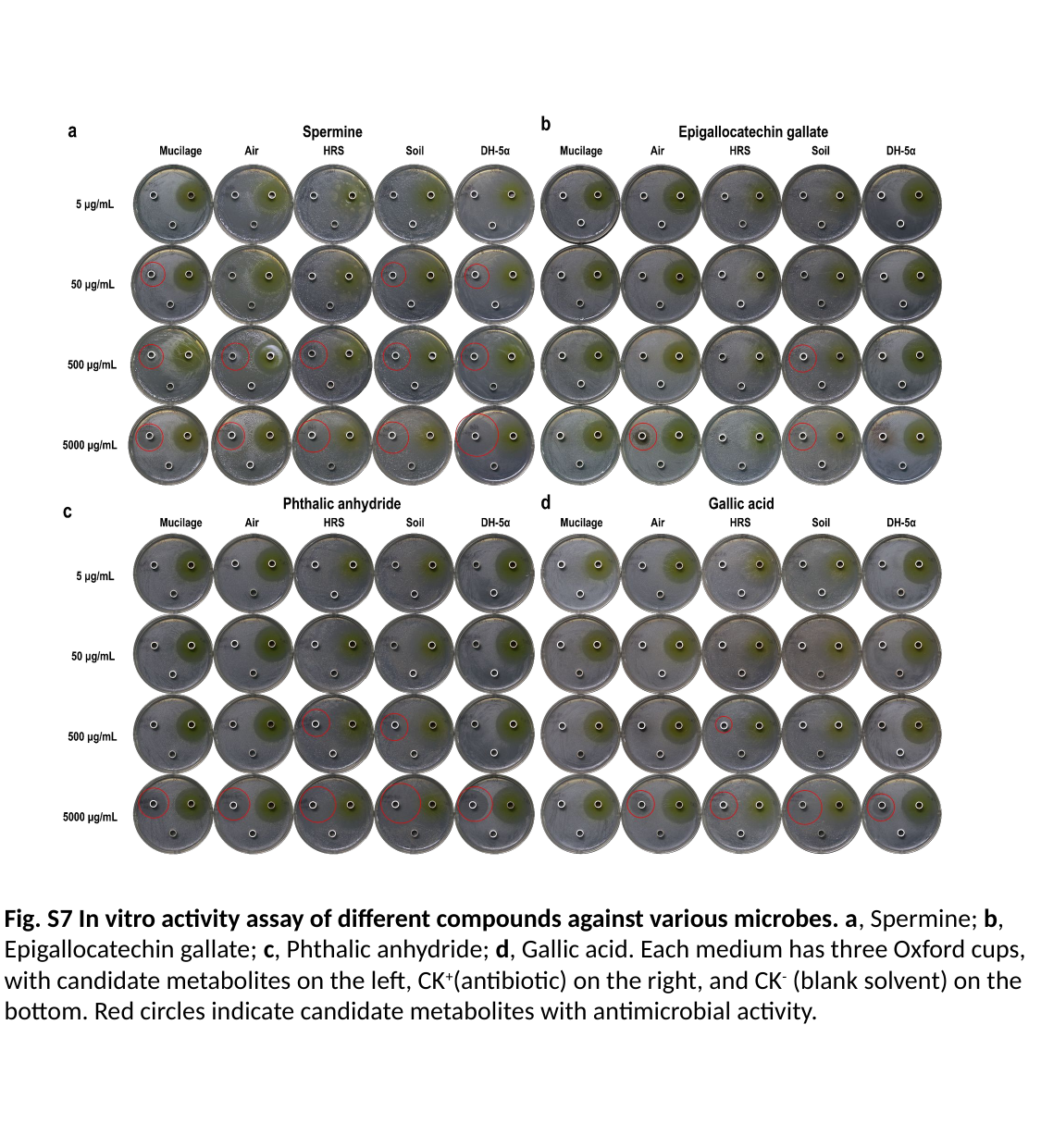

Fig. S7 In vitro activity assay of different compounds against various microbes. a, Spermine; b, Epigallocatechin gallate; c, Phthalic anhydride; d, Gallic acid. Each medium has three Oxford cups, with candidate metabolites on the left, CK+(antibiotic) on the right, and CK- (blank solvent) on the bottom. Red circles indicate candidate metabolites with antimicrobial activity.

## Slide 8
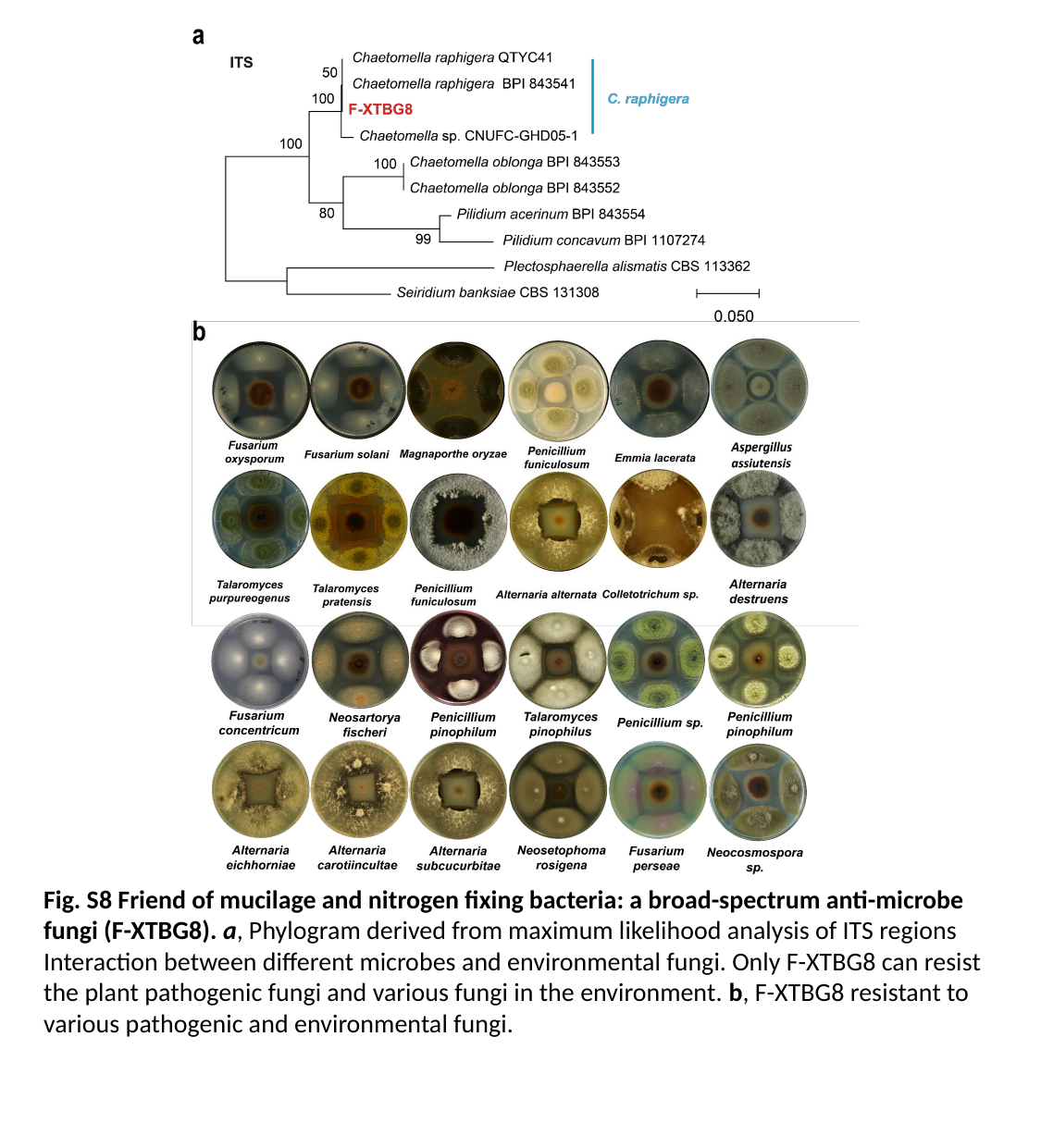

Fig. S8 Friend of mucilage and nitrogen fixing bacteria: a broad-spectrum anti-microbe fungi (F-XTBG8). a, Phylogram derived from maximum likelihood analysis of ITS regions Interaction between different microbes and environmental fungi. Only F-XTBG8 can resist the plant pathogenic fungi and various fungi in the environment. b, F-XTBG8 resistant to various pathogenic and environmental fungi.

## Slide 9
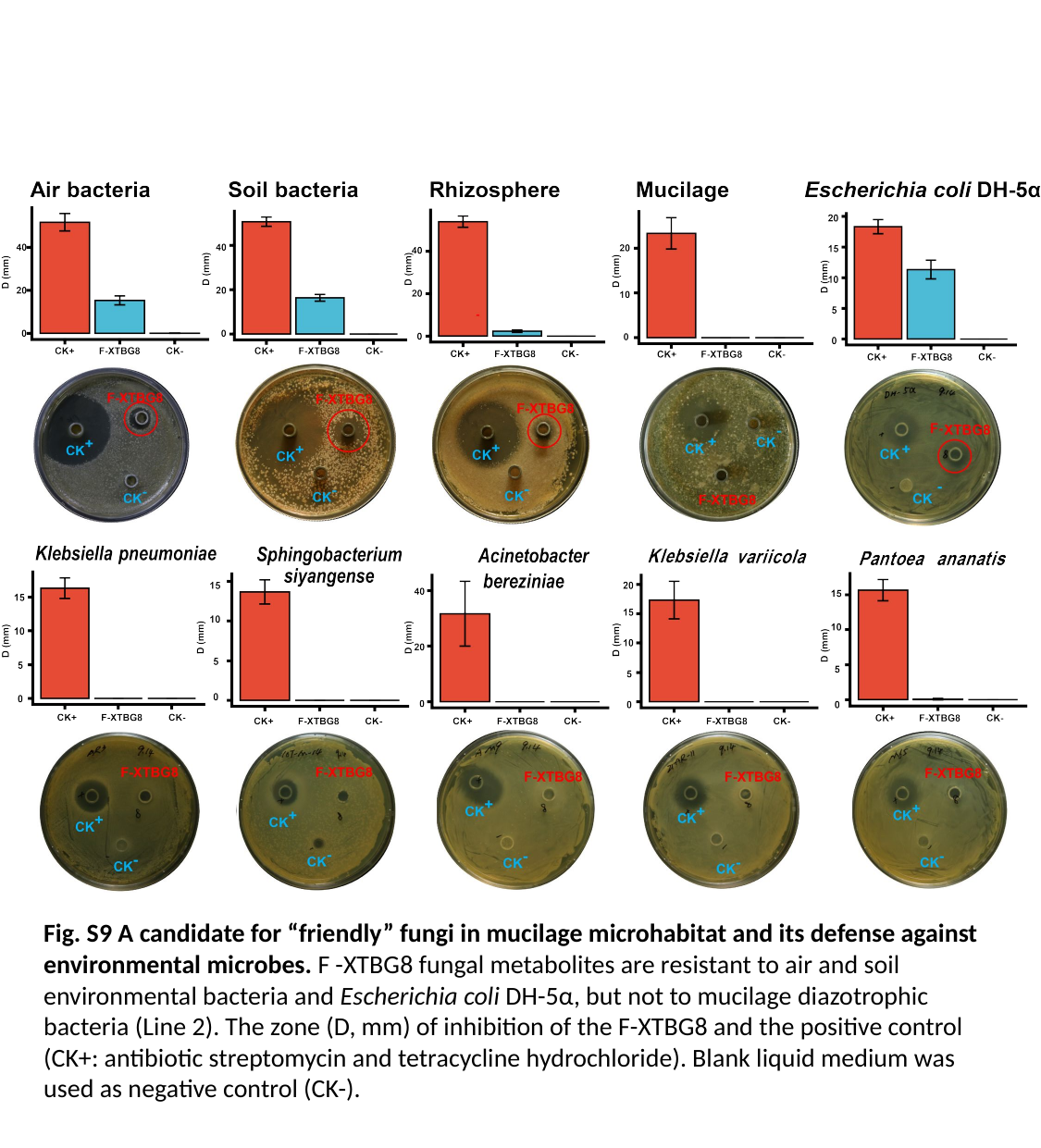

Fig. S9 A candidate for “friendly” fungi in mucilage microhabitat and its defense against environmental microbes. F -XTBG8 fungal metabolites are resistant to air and soil environmental bacteria and Escherichia coli DH-5α, but not to mucilage diazotrophic bacteria (Line 2). The zone (D, mm) of inhibition of the F-XTBG8 and the positive control (CK+: antibiotic streptomycin and tetracycline hydrochloride). Blank liquid medium was used as negative control (CK-).

## Slide 10
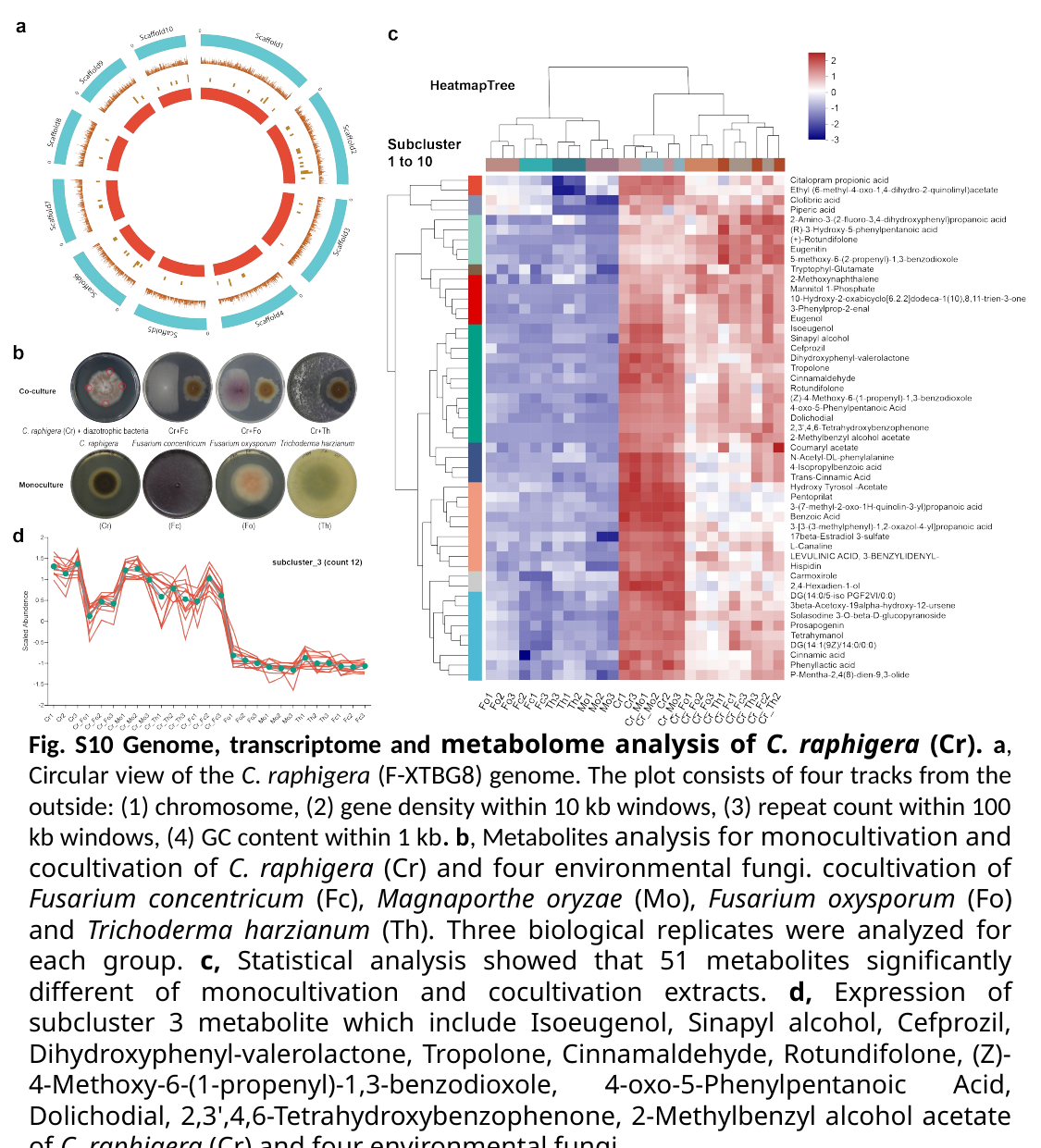

Fig. S10 Genome, transcriptome and metabolome analysis of C. raphigera (Cr). a, Circular view of the C. raphigera (F-XTBG8) genome. The plot consists of four tracks from the outside: (1) chromosome, (2) gene density within 10 kb windows, (3) repeat count within 100 kb windows, (4) GC content within 1 kb. b, Metabolites analysis for monocultivation and cocultivation of C. raphigera (Cr) and four environmental fungi. cocultivation of Fusarium concentricum (Fc), Magnaporthe oryzae (Mo), Fusarium oxysporum (Fo) and Trichoderma harzianum (Th). Three biological replicates were analyzed for each group. c, Statistical analysis showed that 51 metabolites significantly different of monocultivation and cocultivation extracts. d, Expression of subcluster 3 metabolite which include Isoeugenol, Sinapyl alcohol, Cefprozil, Dihydroxyphenyl-valerolactone, Tropolone, Cinnamaldehyde, Rotundifolone, (Z)-4-Methoxy-6-(1-propenyl)-1,3-benzodioxole, 4-oxo-5-Phenylpentanoic Acid, Dolichodial, 2,3',4,6-Tetrahydroxybenzophenone, 2-Methylbenzyl alcohol acetate of C. raphigera (Cr) and four environmental fungi.
