# Supplementary material for: Microbiota-mediated nitrogen fixation and microhabitat homeostasis in aerial root-mucilage
Source: Microbiome. 2023 Apr 21;11:85. doi: 10.1186/s40168-023-01525-x (PMC10120241; doi:10.1186/s40168-023-01525-x)
Supplement: Supplementary file 4 — Additional file 3: Method S1. Carbohydrate and widely targeted metabolites profiling. Method S2. Plant Genome and transcriptome analysis. [file 40168_2023_1525_MOESM3_ESM.zip › Supplemental Material.pdf]

Additional files 1 (Supplementary Method 1-2)

Supplementary Methods 1: Carbohydrate and widely targeted metabolites profiling.

1.1 Widely targeted metabolites profiling.

1.1.1 Sample preparation and extraction (Root exudates)

(1) After thawing the samples from the refrigerator at -80°C, mix them with vortex for 10 s.

(2) Take 40mL of the sample after mixing, place 50mL of the centrifuge tube, and then immerse the sample in liquid nitrogen as a whole. Put the sample into the lyophilizer for freeze-drying after the sample is completely frozen.

(3) After the samples were completely lyophilized, 1ml 70% methanol internal standard extract was added. Scroll for 3 min。

(4) Centrifuge (12000 r/min, 4°C) for 10min. The supernatant was filtered with a microporous filter membrane (0.22 m) and stored in a sample flask for LC-MS/MS test.

1.1.2 UPLC Conditions

The sample extracts were analyzed using an UPLC-ESI-MS/MS system (UPLC, SHIMADZU Nexera X2, https://www.shimadzu.com.cn/; MS, Applied Biosystems 4500 Q TRAP, https://www.thermofisher.cn/cn/zh/home/brands/applied-biosystems.html). The analytical conditions were as follows, UPLC: column, Agilent SB-C18 (1.8 µm, 2.1 mm * 100 mm); The mobile phase was consisted of solvent A, pure water with 0.1% formic acid, and solvent B, acetonitrile with 0.1% formic acid. Sample measurements were performed with a gradient program that employed the starting conditions of 95% A, 5% B. Within 9 min, a linear gradient to 5% A, 95% B was programmed, and a composition of 5% A, 95% B was kept for 1 min. Subsequently, a composition of 95% A, 5.0% B was adjusted within 1.1 min and kept for 2.9 min.

The flow velocity was set as 0.35 mL per minute; The column oven was set to 40°C; The injection volume was 4 μL. The effluent was alternatively connected to an ESI-triple quadrupole-linear ion trap (QTRAP)-MS.

1.1.3 ESI-Q TRAP-MS/MS

LIT and triple quadrupole (QQQ) scans were acquired on a triple quadrupole-linear ion trap mass spectrometer (Q TRAP), AB4500 Q TRAP UPLC/MS/MS System, equipped with an ESI Turbo Ion-Spray interface, operating in positive and negative ion mode and controlled by Analyst 1.6.3 software (AB Sciex). The ESI source operation parameters were as follows: ion source, turbo spray; source temperature 550°C; ion spray voltage (IS) 5500 V (positive ion mode)/-4500 V (negative ion mode); ion source gas I (GSI), gas II(GSII), curtain gas (CUR) were set at 50, 60, and 25.0 psi, respectively; the collision-activated dissociation(CAD) was high. Instrument tuning and mass calibration were performed with 10 and 100 μmol/L polypropylene glycol solutions in QQQ and LIT modes, respectively. QQQ scans were acquired as MRM experiments with collision gas (nitrogen) set to medium. DP and CE for individual MRM transitions was done with further DP and CE optimization. A specific set of MRM transitions were monitored for each period according to the metabolites eluted within this period.

1.2 Carbohydrate metabolites profiling

1.2.1 Chemicals and reagents

Methanol (MeOH) were purchased from Merck (Darmstadt, Germany). MilliQ water (Millipore, Bradford, USA) was used in all experiments. All of the standards were purchased from CNW (Shanghai Anpel), IsoReag (Shanghai) and TCI (Shanghai). The stock solutions of standards were prepared at the concentration of 2 mg/mL in MeOH. All stock solutions were stored at -20°C. The stock solutions were diluted with MeOH to working solutions before analysis.

1.2.2 Sample preparation and extraction

The freeze-dried materials were crushed using a mixer mill (MM 400, Retsch) with a zirconia bead for 1.5 min at 30 Hz. 20 mg of powder was diluted to a 500 μL with methanol: isopropanol: water (3:3:2 V/V/V), vortexed for 3 min and ultrasound for 30 min. The extract was centrifuged at 14,000 rpm under 4°C for 3 min. 50μL of the supernatant was mixed with 20 μL internal standard (ribitol, 100 μg/mL) and evaporated under nitrogen gas stream. The evaporated sample was transferred to the lyophilizer for freeze-drying. The residue was used for the further derivatization. The derivatization method was as follows: the sample was mixed with 100 μL solution of methoxyamine hydrochloride in pyridine (15 mg/mL). The mixture was incubated at 37°C for 2 h. Then 100 μL of BSTFA was added into the mixture and kept at 37°C for 30 min after vortex-mixing. The mixture was analyzed by GC-MS after diluting to an appropriate concentration.

1.2.3 GC-MS analysis

Agilent 7890B gas chromatograph coupled to a 7000D mass spectrometer with a DB-5MS column (30 m length × 0.25 mm i.d. × 0.25 μm film thickness, J&W Scientific, USA) was employed for GC-MS analysis of sugars. Helium was used as carrier gas, at a flow rate of 1 mL/min. Injections were made in the split mode with a split ratio 3:1 and the intection volume was 3 μL. The oven temperature was held at 170°C for 2min, and then raised to 240°C at 10°C/min , raised to 280°C at 5°C/min , raised to 310°C at 25°C/min and held at the temperature for 4 min. All samples were analyzed in selective ion monitoring mode. The ion source and transfer line temperature were 230°C and 240°C, respectively.

Supplementary Methods 2: *H. rotundifolia* Genome and transcriptome analysis

2.1 Sample and sequencing

For genome sequencing, we collected dozens of *H. rotundifolia* plants from Xishuangbanna Tropical Botanical Garden (Xishuangbanna, China). Fresh and healthy *H. rotundifolia* were harvested from the best-growing individual and immediately frozen in liquid nitrogen, followed by

preservation at −80 °C in the laboratory prior to DNA extraction. High-quality genomic DNA was extracted from leaves using QIAGEN DNaesy Plant Mini Kit and according to the manufacturer's protocols. RNase A was used to remove RNA contaminants. The quality of the DNA was checked by agarose gel electrophoresis, and an excellent integrity of DNA molecules were observed. The extracted DNA molecules were sequenced by PacBio Sequel (Pacific Biosciences of California, Menlo Park, CA, USA) platforms.

2.2 Genome assembly by third-generation long reads

The draft assembly of the genome is assembled using mecat2 (20190226) with default parameters. To correct errors in the primary assembly, we used the arrow pipeline from the SMRT link 4 tool kit to polish the genome (https://www.pacb.com/products-and-services/analytical-software/smrt-analysis/) after the initial assembly of the genome was completed. Finally, we used Illumina-derived short reads to correct any remaining errors by pilon (v1.22) [1].

2.3 Hi-C technology help anchor contigs

Hi-C reads were mapped to the polished *H. rotundifolia* genome using BWA (bwa-0.7.17) with the default parameters [2]. Paired reads with mate mapped to a different contig were used to do the Hi-C associated scaffolding. Self-ligation, non-ligation and other invalid reads, such as Start NearRsite, PCR amplification, random break, Large Smal lFragments and Extreme Fragments, were filtered. Lachesis was further applied to order and orient the clustered contigs.

2.4 Annotation of repetitive sequences

The two methods are combined to identify the repeat contents in our genome, homology-based and de novo prediction. Homology-based analysis: We identified the known TEs within the *H. rotundifolia* genome using RepeatMasker (open-4.0.9) [3] with the Repbase TE library [4]. RepeatProteinMask searches were also conducted using the TE protein database as a query library. De novo prediction: We constructed a de novo repeat library of the *H. rotundifolia* genome using RepeatModeler (http://www.repeatmasker.org/RepeatModeler/), which can automatically execute two core de novo repeat-finding programs, namely, RECON (v1.08) [5] and RepeatScout

(v1.0.5) [6], to comprehensively conduct, refine and classify consensus models of putative interspersed repeats for the *H. rotundifolia* genome. Furthermore, we performed a de novo search for long terminal repeat [7] retrotransposons against the *H. rotundifolia* genome sequences using LTR_FINDER (v1.0.7) [8]. We also identified tandem repeats using the Tandem Repeat Finder (TRF) package [9] and the non-interspersed repeat sequences, including low-complexity repeats, satellites and simple repeats, using RepeatMasker. Finally, we merge the lib library files of the two methods and use repeat maker to identify the repeat contents.

2. 5 Annotation of Protein coding gene

We predicted protein-coding genes of the *H. rotundifolia* genome using three methods, including ab initio gene prediction, homology-based gene prediction and RNA-Seq-aided gene prediction. Prior to gene prediction, the assembled *H. rotundifolia* genome was hard and soft masked using RepeatMasker. We adopted Augustus (v3.3.1) [10-12] and Genescan [13] to perform ab initio gene prediction. Models used for each gene predictor were trained from a set of high-quality proteins generated from the RNA-Seq dataset. We used Exonerate (v2.2.0) [14] to conduct homology-based gene prediction. First, the protein sequences were aligned to our genome assembly and predicted coding gene using Exonerate with the default parameters. To carry out RNA-Seq-aided gene prediction, we first assembled clean RNA-Seq reads into transcripts using TopHat (v2.1.1) [15], and the gene structure were formed using Cufflinks (v2.2.1) [16]. Finally, Maker (v3.00) [17] was used to integrate the prediction results of the three methods to predict genes modles. The output included a set of consistent and non-overlapping sequence assemblies, which were used to describe the gene structures. In total, 41,252 protein-coding genes with an average length of 5,639 bp were predicted in the assembled *H. rotundifolia* genome.

2. 6 Functional annotation of protein-coding genes

Gene functions were inferred according to the best match of the alignments to the National Center for Biotechnology Information (NCBI) Non-Redundant (NR), TrEMBL [18], InterPro [19] and Swiss-Prot protein databases using BLASTP (ncbi blast v2.6.0+) [20,21] and the Kyoto Encyclopedia of Genes and Genomes (KEGG) database [22] with an E-value threshold of 1E-5. The protein

domains were annotated using PfamScan (pfamscan_version) [23] and InterProScan (v5.35-74.0) [24] based on InterPro protein databases. The motifs and domains within gene models were identified by PFAM databases. Gene Ontology (GO) [25] IDs for each gene were obtained from Blast2GO [26]. In total, approximately 39,068 (about 94.71%) of the predicted protein-coding genes of *H. rotundifolia* could be functionally annotated with known genes, conserved domains, and Gene Ontology terms.

2. 7 Annotation of non-coding RNA genes

We used tRNAscan-SE (v1.3.1) algorithms [27] with default parameters to identify the genes associated with tRNA, which is an adaptor molecule composed of RNA used in biology to bridge the three-letter genetic code in messenger RNA (mRNA) with the twenty-letter code of amino acids in proteins. For rRNA identification, we first downloaded the closely related species rRNA sequences from the Ensembl database. Then rRNAs in the database were aligned against our genome using blastn [20] with cutoff of E-value <1e-5, identity ≥85% and match length ≥ 50 bp. snoRNAs are a class of small RNA molecules that guide chemical modifications of other RNAs, mainly ribosomal RNAs, transfer RNAs and small nuclear RNAs. MiRNAs and snRNAs were identified by Infernal (v1.1.2) [28] software against the Rfam (v14.1) database [29] with default parameters.

2. 8 *H. rotundifolia* RNA isolation and transcriptome analysis

Total RNA was extracted using Trizol reagent following manufacturer’s recommendations (Invitrogen, CA, USA). RNA purity and integrity was assessed using NanoDrop 2000 spectrophotometer (NanoDrop Technologies, Wilmington, DE, USA) and Bioanalyzer 2100 system (Agilent Technologies, CA, USA). RNA contamination was assessed by 1.5% agarose gel electrophoresis. A total of 1 μg RNA per sample was used as the input material for library preparation. The mRNA was purified from the total RNA using poly - Toligo - attached magnetic beads. Sequencing libraries were generated from the purified mRNA using the VAHTS Universal V6 RNA-seq Library Kit for MGI (Vazyme, Nanjing, China) following the manufacturer's recommendations with unique index codes. The Library quantification and size was assessed

using Qubit 3.0 Fluorometer (Life Technologies, Carlsbad, CA, USA) and Bioanalyzer 2100 system (Agilent Technologies, CA, USA). Subsequently, sequencing was performed on a MGI-SEQ 2000 platform by Frasergen Bioinformatics Co., Ltd. (Wuhan, China). Low quality reads were filtered out by SOAPnuke software [30] and clean reads were mapped to *H. rotundifolia* genome using bowtie2 software [31]. Gene expression levels were estimated using FPKM values (fragments per kilobase per million fragments mapped) by the RSEM software [32]. DESeq2 [33] was used to evaluate differential expression genes between different root samples. Genes with fold change > 1 or < -1, and FDR < 0.05 were differential expressed genes.

1 Walker, B. J. *et al.* Pilon: an integrated tool for comprehensive microbial variant detection and genome assembly improvement. *PLoS One* 9, e112963, doi:10.1371/journal.pone.0112963 (2014).

2 Burton, J. N. *et al.* Chromosome-scale scaffolding of de novo genome assemblies based on chromatin interactions. *Nat Biotechnol* 31, 1119-1125, doi:10.1038/nbt.2727 (2013).

3 Tarailo-Graovac, M. & Chen, N. Using RepeatMasker to identify repetitive elements in genomic sequences. *Current protocols in bioinformatics* Chapter 4, Unit 4.10, doi:10.1002/0471250953.bi0410s25 (2009).

4 Jurka, J. *et al.* Repbase Update, a database of eukaryotic repetitive elements. *Cytogenetic and genome research* 110, 462-467, doi:10.1159/000084979 (2005).

5 Bao, Z. & Eddy, S. R. Automated de novo identification of repeat sequence families in sequenced genomes. *Genome Res* 12, 1269-1276, doi:10.1101/gr.88502 (2002).

6 Price, A. L., Jones, N. C. & Pevzner, P. A. De novo identification of repeat families in large genomes. *Bioinformatics* 21, i351-i358, doi:10.1093/bioinformatics/bti1018 %J Bioinformatics (2005).

7 Baltrus, D. A. Bacterial dispersal and biogeography as underappreciated influences on phytobiomes. *Current Opinion in Plant Biology* 56, 37-46, doi:10.1016/j.pbi.2020.02.010 (2020).

8 Xu, Z. & Wang, H. LTR_FINDER: an efficient tool for the prediction of full-length LTR retrotransposons. *Nucleic acids research* 35, W265-W268, doi:10.1093/nar/gkm286 (2007).

9 Benson, G. Tandem repeats finder: a program to analyze DNA sequences. *Nucleic Acids Res* 27, 573-580, doi:10.1093/nar/27.2.573 (1999).

10 Stanke, M., Steinkamp, R., Waack, S. & Morgenstern, B. AUGUSTUS: a web server for gene finding in eukaryotes. *Nucleic Acids Res* 32, W309-312, doi:10.1093/nar/gkh379 (2004).

11 Stanke, M. & Morgenstern, B. AUGUSTUS: a web server for gene prediction in eukaryotes that allows user-defined constraints. *Nucleic Acids Res* 33, W465-467, doi:10.1093/nar/gki458 (2005).

12 Stanke, M. *et al.* AUGUSTUS: ab initio prediction of alternative transcripts. *Nucleic Acids Res* 34, W435-439, doi:10.1093/nar/gkl200 (2006).

13 Burge, C. & Karlin, S. Prediction of complete gene structures in human genomic DNA. *Journal of molecular biology* 268, 78-94, doi:10.1006/jmbi.1997.0951 (1997).

14 Slater, G. S. C. & Birney, E. Automated generation of heuristics for biological sequence comparison. *BMC Bioinformatics* 6, 31, doi:10.1186/1471-2105-6-31 (2005).

15 Trapnell, C., Pachter, L. & Salzberg, S. L. TopHat: discovering splice junctions with RNA-Seq.

Bioinformatics 25, 1105-1111, doi:10.1093/bioinformatics/btp120 (2009).
16 Trapnell, C. *et al.* Transcript assembly and quantification by RNA-Seq reveals unannotated transcripts and isoform switching during cell differentiation. *Nat Biotechnol* 28, 511-515, doi:10.1038/nbt.1621 (2010).
17 Cantarel, B. L. *et al.* MAKER: an easy-to-use annotation pipeline designed for emerging model organism genomes. *Genome Res* 18, 188-196, doi:10.1101/gr.6743907 (2008).
18 Boeckmann, B. *et al.* The SWISS-PROT protein knowledgebase and its supplement TrEMBL in 2003. *Nucleic Acids Research* 31, 365-370, doi:10.1093/nar/gkg095 %J Nucleic Acids Research (2003).
19 Mitchell, A. *et al.* The InterPro protein families database: the classification resource after 15 years. *Nucleic acids research* 43, D213-221, doi:10.1093/nar/gku1243 (2015).
20 Altschul, S. F. *et al.* Gapped BLAST and PSI-BLAST: a new generation of protein database search programs. *Nucleic Acids Res* 25, 3389-3402, doi:10.1093/nar/25.17.3389 (1997).
21 Camacho, C. *et al.* BLAST+: architecture and applications. *BMC Bioinformatics* 10, 421, doi:10.1186/1471-2105-10-421 (2009).
22 Kanehisa, M., Goto, S., Sato, Y., Furumichi, M. & Tanabe, M. KEGG for integration and interpretation of large-scale molecular data sets. *Nucleic Acids Res* 40, D109-114, doi:10.1093/nar/gkr988 (2012).
23 Mistry, J., Bateman, A. & Finn, R. D. Predicting active site residue annotations in the Pfam database. *BMC bioinformatics* 8, 298-298, doi:10.1186/1471-2105-8-298 (2007).
24 Jones, P. *et al.* InterProScan 5: genome-scale protein function classification. *Bioinformatics* 30, 1236-1240, doi:10.1093/bioinformatics/btu031 (2014).
25 Ashburner, M. *et al.* Gene Ontology: tool for the unification of biology. *Nature Genetics* 25, 25-29, doi:10.1038/75556 (2000).
26 Conesa, A. & Götz, S. Blast2GO: A comprehensive suite for functional analysis in plant genomics. *Int J Plant Genomics* 2008, 619832-619832, doi:10.1155/2008/619832 (2008).
27 Lowe, T. M. & Eddy, S. R. tRNAscan-SE: a program for improved detection of transfer RNA genes in genomic sequence. *Nucleic Acids Res* 25, 955-964, doi:10.1093/nar/25.5.955 (1997).
28 Nawrocki, E. P., Kolbe, D. L. & Eddy, S. R. Infernal 1.0: inference of RNA alignments. *Bioinformatics* 25, 1335-1337, doi:10.1093/bioinformatics/btp157 (2009).
29 Bateman, A. *et al.* The Pfam protein families database. *Nucleic acids research* 28, 263-266, doi:10.1093/nar/28.1.263 (2000).
30 Chen, Y. *et al.* SOAPnuke: a MapReduce acceleration-supported software for integrated quality control and preprocessing of high-throughput sequencing data. *GigaScience* 7, doi:10.1093/gigascience/gix120 (2017).
31 Langmead, B. Aligning short sequencing reads with Bowtie. *Current protocols in bioinformatics* Chapter 11, Unit 11.17, doi:10.1002/0471250953.bi1107s32 (2010).
32 Li, B. & Dewey, C. N. RSEM: accurate transcript quantification from RNA-Seq data with or without a reference genome. *BMC Bioinformatics* 12, 323, doi:10.1186/1471-2105-12-323 (2011).
33 Love, M. I., Huber, W. & Anders, S. Moderated estimation of fold change and dispersion for RNA-seq data with DESeq2. *Genome Biol* 15, 550, doi:10.1186/s13059-014-0550-8 (2014).
